# Comparative proteomic analysis of pathogenic and non-pathogenic strains from the swine pathogen *Mycoplasma hyopneumoniae*

**DOI:** 10.1186/1477-5956-7-45

**Published:** 2009-12-21

**Authors:** Paulo M Pinto, Cátia S Klein, Arnaldo Zaha, Henrique B Ferreira

**Affiliations:** 1 Laboratório de Genômica Estrutural e Funcional, Centro de Biotecnologia, UFRGS, Porto Alegre, RS, Brazil; 2 Current address: Instituto de Biotecnologia, Universidade de Caxias do Sul, Caxias do Sul, RS, Brazil; 3 CNPSA/EMBRAPA, Concórdia, SC, Brazil; 4 Departamento de Biologia Molecular e Biotecnologia, Instituto de Biociências, UFRGS, Porto Alegre, RS, Brazil

## Abstract

**Background:**

*Mycoplasma hyopneumoniae *is a highly infectious swine pathogen and is the causative agent of enzootic pneumonia (EP). Following the previous report of a proteomic survey of the pathogenic 7448 strain of swine pathogen, *Mycoplasma hyopneumoniae*, we performed comparative protein profiling of three *M. hyopneumoniae *strains, namely the non-pathogenic J strain and the two pathogenic strains 7448 and 7422.

**Results:**

In 2DE comparisons, we were able to identify differences in expression levels for 67 proteins, including the overexpression of some cytoadherence-related proteins only in the pathogenic strains. 2DE immunoblot analyses allowed the identification of differential proteolytic cleavage patterns of the P97 adhesin in the three strains. For more comprehensive protein profiling, an LC-MS/MS strategy was used. Overall, 35% of the *M. hyopneumoniae *genome coding capacity was covered. Partially overlapping profiles of identified proteins were observed in the strains with 81 proteins identified only in one strain and 54 proteins identified in two strains. Abundance analysis of proteins detected in more than one strain demonstrates the relative overexpression of 64 proteins, including the P97 adhesin in the pathogenic strains.

**Conclusions:**

Our results indicate the physiological differences between the non-pathogenic strain, with its non-infective proliferate lifestyle, and the pathogenic strains, with its constitutive expression of adhesins, which would render the bacterium competent for adhesion and infection prior to host contact.

## Background

*Mycoplasma hyopneumoniae *is a highly infectious swine pathogen and is the causative agent of enzootic pneumonia (EP), a disease characterised by a sporadic, dry, and non-productive cough, retarded growth, and inefficient food conversion [1]. Despite presenting with low direct mortality rates, EP is responsible for major economic losses in the pig industry as it causes increased susceptibility to secondary respiratory infections due to the *M. hyopneumoniae*-associated deactivation of mucociliary functions. The *M. hyopneumoniae *genomes of a non-pathogenic (J) and two pathogenic strains (strains 7448 and 232) [2,3] have been sequenced. Comparative genomic analyses provided insights into evolutionary aspects of mycoplasma reduced genomes [4] and *M. hyopneumoniae *virulence determinants [5,6].

Thus far, the study of virulence factors in *M. hyopneumoniae *has been focused on the characterisation of adhesion mediating molecules, especially the P97 adhesin [7,8]. However, the mechanisms of *M. hyopneumoniae *pathogenicity suggest the existence of several other classes of unidentified virulence factors, such as genes/proteins involved in secretion and/or trafficking of molecules between host and pathogen cells, or evasion and/or modulation of the host immune system [6]. The observed presence of a putative integrative conjugal element (ICEH) in three *M. hyopneumoniae *pathogenic strains, but not in a non-pathogenic strain, suggests the involvement of potentially mobile genetic elements in the *M. hyopneumoniae *virulence [6,9].

In many species, differences in pathogenicity or other biological features between strains correspond to significant differences at the genomic level. For example, speciation and diversity of strains have been achieved by horizontal gene transfer of DNA encoding novel genes that are likely to be required for niche specific survival [10]. In the case of *M. hyopneumoniae*, no extensive genomic differences have been detected among the genomes of different strains, even in the comparison between the non-pathogenic strain J and the pathogenic strains 7448 and 232 [2,5]. In this context, it can be assumed that differences between pathogenic determinants are not predominantly at the genomic level. Rather, these differences may be associated with variations in expression levels of genes encoding virulence factors. Therefore, comparative transcriptomic and proteomic analyses of relevant strains have the potential to discover gene products that play a role in *M. hyopneumoniae *pathogenesis.

Transcriptomic studies aimed at identifying differentially expressed genes have recently been published [11-15]. However, these studies were unable to identify genes specifically related to virulence, even when analysing the infection conditions [13]. The *M. hyopneumoniae *proteomic studies performed thus far were mainly prospective. Results included the 2DE mapping of the *M. hyopneumoniae *strain 7448 [16] as well as evidence of post-translational modifications of several *M. hyopneumoniae *proteins, including P97 [8,16]. A recent comparative proteomic report of the *M. hyopneumoniae *J and 232 strains based on 2DE and mass spectrometry analyses showed at least 10 proteins with differential expression levels [17]. More comprehensive and comparative proteomic approaches are expected to provide an overview of the *M. hyopneumoniae *repertoire of virulence-related proteins.

In this study, we compare the proteomes of the avirulent J strain and the virulent 7422 and 7448 strains using a liquid chromatography separation coupled with tandem mass spectrometry (LC-MS/MS) approach. We experimentally confirmed the expression of 231 *M. hyopneumoniae *protein gene products, which represents approximately 35% of the genome coding capacity of this bacterium. Relative abundance data were also provided for these proteins based on the exponentially modified protein abundance index (emPAI). This survey and complementary 2DE and immunoblotting analyses constituted the first comprehensive comparative proteomic analysis of *M. hyopneumoniae *strains and demonstrate the overexpression of virulence factors specifically in the pathogenic strains.

## Results

### 2DE comparative analysis of *M. hyopneumoniae *strains J, 7448, and 7422

For a comparative investigation of the repertoires of protein gene products in *M. hyopneumoniae *strains J, 7448, and 7422, the respective protein extracts were analysed by 2DE. In each gel, proteins were identified by matching the respective spots to the previously established proteomic maps of *M. hyopneumoniae *7448 [16]. The average spot matching rate for the replicate gels of each sample was 0.92, meaning that 92% of the spots in each gel were also found in the corresponding replicates, thereby validating the 2DE results as reproducible and reliable.

**Figure 1:**
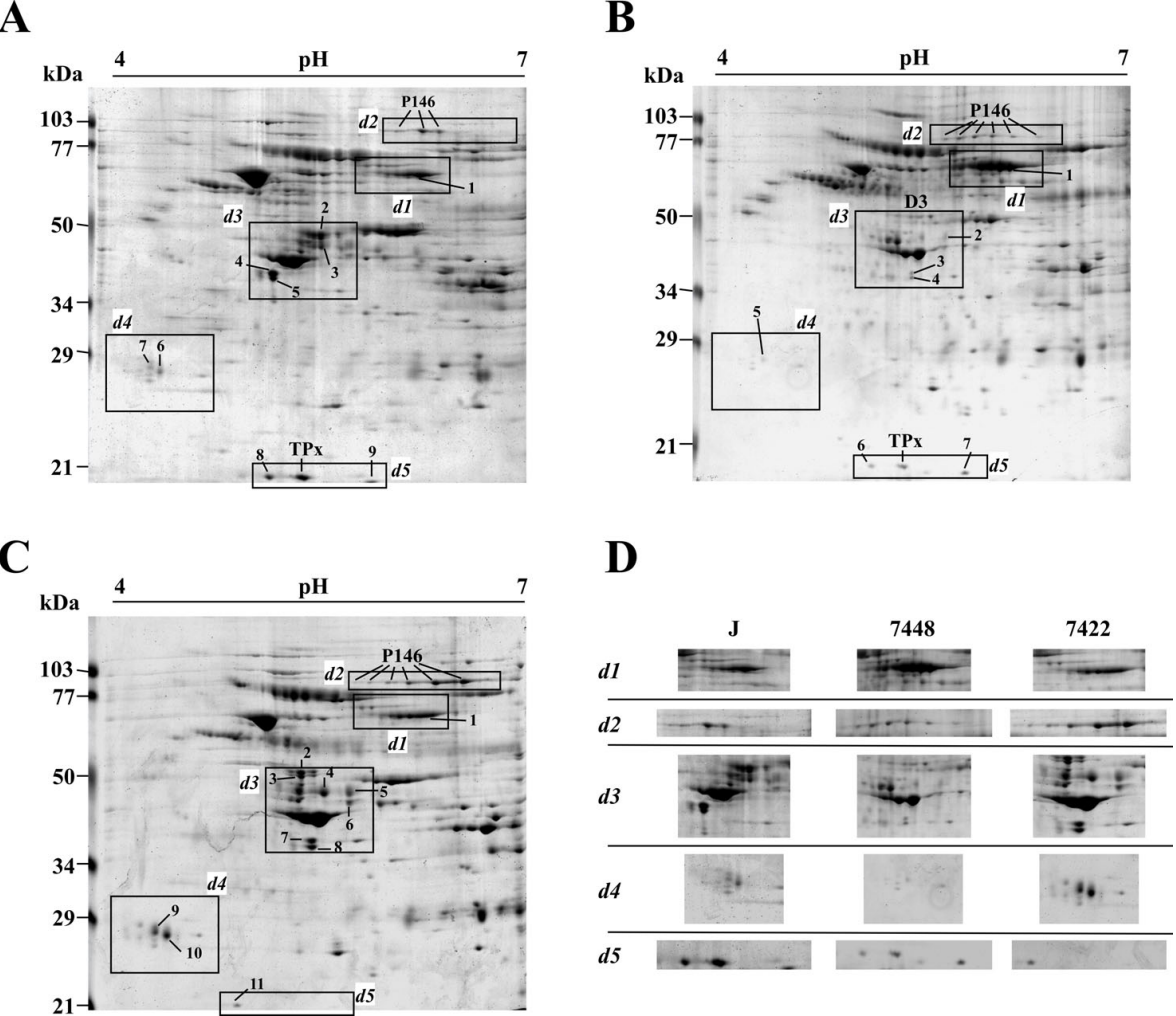
**2DE proteome profiling the three *M. hyopneumoniae *strains with IEF at pH 4-7**. Protein samples (2 mg) from the *M. hyopneumoniae *strains J (A), 7448 (B), and 7422 (C) were separated by IEF using 17 cm pH 4-7 IPG strips, followed by SDS-PAGE on 12% gels and stained with Coomassie Brilliant Blue G. The approximate molecular weights are shown on the left of the gel and the acid-to-alkaline gradient is from left to right. The rectangle delimited areas (numbered *d1-d5*) in the gels and panels in (D) show gel regions in which spots corresponding to differentially expressed proteins were identified. Spots corresponding to proteins identified by matching to the previously reported *M. hyopneumoniae *7448 proteome maps [16] were named according to the predicted gene products. Spots corresponding to proteins thus far unidentified in proteome maps were numbered.

**Figure 2:**
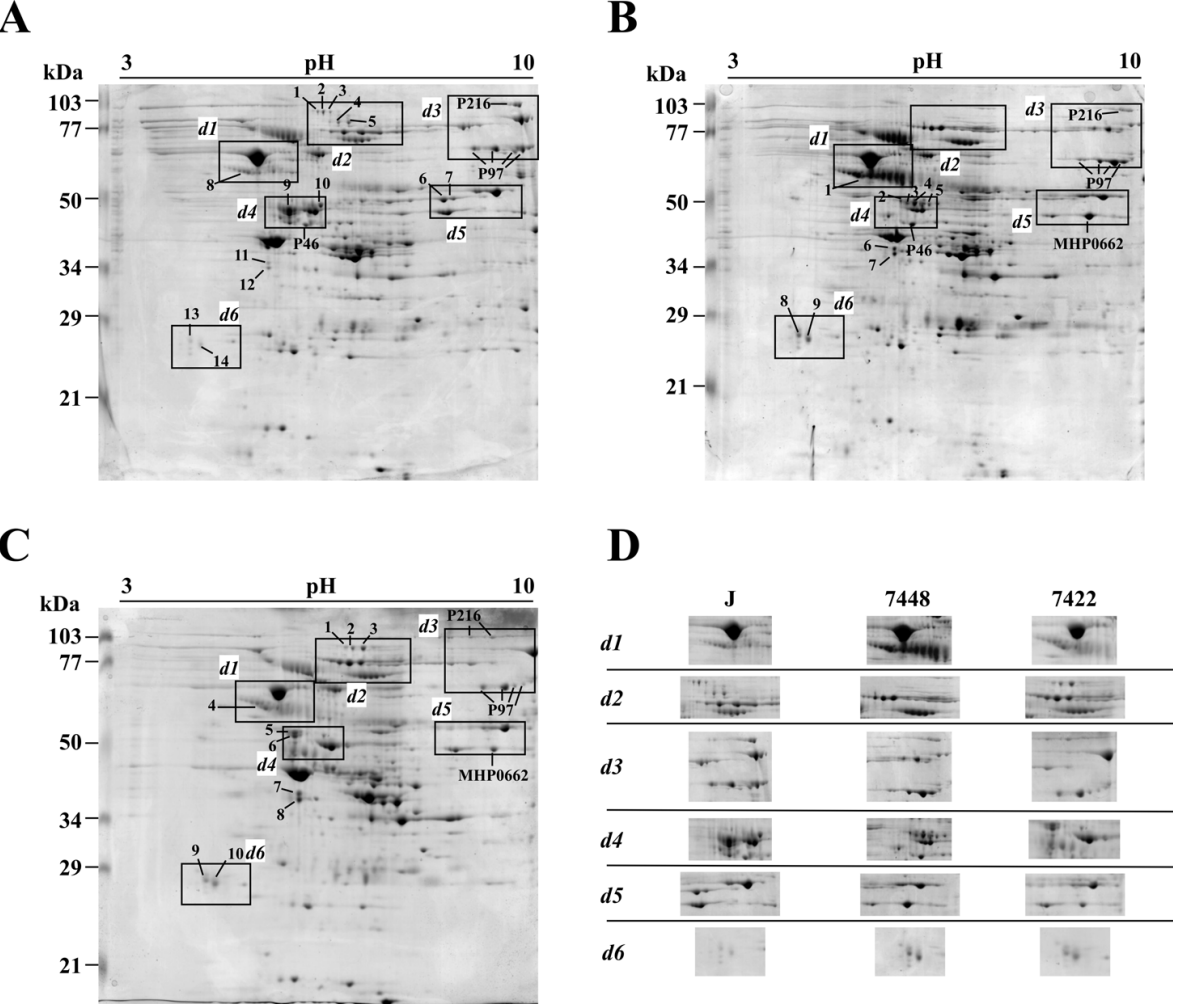
**2DE proteome profiling of the three *M. hyopneumoniae *strains with IEF at pH 3-10**. Protein samples (2 mg) from the three *M. hyopneumoniae *strains J (A), 7448 (B), and 7422 (C) were separated by IEF using 17 cm pH 3-10 IPG strips, followed by SDS-PAGE on 12% gels and stained with Coomassie brilliant blue G. The approximate molecular weights are shown on the left of the gel and the acid-to-alkaline gradient is from left to right. The rectangle delimited areas (numbered *d1-d6*) in the gels and panels in (D) show gel regions in which spots corresponding to differentially expressed proteins were identified. Spots corresponding to proteins identified by matching to the previously reported *M. hyopneumoniae *7448 proteome maps [16] were named according to the predicted gene products or by the corresponding CDS number. Spots corresponding to proteins thus far unidentified in proteome maps were numbered.

Significant qualitative (presence versus absence) or quantitative differences between the three *M. hyopneumoniae *strains under the same culture conditions were identified including 38 spots (17 identified, corresponding to 2 different proteins) of 2DE in the pH 4-7 range (Figure 1) and 29 spots (10 identified, corresponding to 4 different proteins) of 2DE in the pH 3-10 range (Figure 2). In the 2DE analyses of pathogenic strains (7448 and 7422), we identified several overexpressed surface and/or cytoadhesion-related proteins (P46, P97, and P146), and a protein previously described as hypothetical (product of the MHP0662 CDS) [2]. The number of P97 and P146 pI isoforms observed in these strains was also distinct, which denotes differences in post-translational processing. Two proteins were identified that showed overexpression in the J strain: 1) P216, an adhesin; and thiol peroxidase (TPx, reannotated as a peroxiredoxin [18]), an oxidative stress-related protein described as a virulence factor for other pathogenic bacteria [19,20].

### Comparative shotgun proteomics of *M. hyopneumoniae*

Since 2DE does not favour the identification of less abundant protein species or proteins with extremes of pI, we performed a complementary shotgun LC-MS/MS analysis for a more comprehensive comparative analysis of the protein expression profiles of the *M. hyopneumoniae *J, 7448, and 7422 strains. Protein extracts from *in vitro *cultured strains were individually digested with trypsin and each of the resulting peptide mixtures were independently analysed three times by LC-MS/MS. A >93% concordance in the repertoire of identified proteins was obtained between the three independent LC-MS/MS analyses of each sample. Combined results from the LC-MS/MS experiments of the three strains resulted in a pool of 231 unique protein identifications (from 953 unique peptide sequences). Totals of 164, 154, and 158 proteins were identified for stains J, 7448, and 7422, respectively (Additional Files 1, 2, 3, 4: Tables S1-S4). The genome-wide coverage of the obtained proteomic data corresponds to 35% of the *M. hyopneumoniae *genome theoretical coding capacity and provided experimental validation for 39 genes previously regarded as hypothetical [2]. By using the target-decoy sequence search strategy, the overall false positive peptide matching rate from the three combined runs is estimated to be 1.27%, 2.13% and 1.72% for J, 7448, and 7422 strains, respectively, which validates our MS/MS results.

In total, 96 of the identified proteins, including 12 products from hypothetical genes, were shared by the three strains. Additionally, 17 proteins were shared by J and 7448 strains, 21 proteins were shared by J and 7422 strains, and 16 proteins were shared by the pathogenic strains, while 81 proteins (more than 35% of the total of identified proteins) were detected in a single strain, with 25 of these single-strain proteins identified in the 7448 strain, 30 in the J strain, and 26 in the 7422 strain. Additional file 1; Table S1 and Additional file 5: Figure S1 summarise the protein sets that are exclusive to one strain, shared by two strains or common to all of them.

To extend our comparative analysis to those proteins shared by two or three strains, we also compared the relative abundance of the LC-MS/MS identified proteins in *M. hyopneumoniae *strains J, 7448, and 7422 using their emPAI values. We were able to infer that the concentration of at least 64 proteins differed significantly among the analysed *M. hyopneumoniae *strains (Additional File 1: Table S1, highlighted in gray, and Additional File 6: Figure S2). In the comparison between the non-pathogenic strain J and the pathogenic strain 7448, 30 proteins were relatively overexpressed in the J strain, while 8 were overexpressed in the 7448 strain. When compared to the 7422 strain, strain J presented 15 proteins as relatively overexpressed, while 19 were classified as overexpressed in the 7442 strain. Finally, in the comparison between the two pathogenic strains, strain 7448 presented 8 proteins with a relative overexpression, and while 27 were classified as overexpressed in the 7442 strain.

In our analyses, repertoires of 3-33 peptides corresponding to the P97 adhesin were identified depending on the strain (Additional Files 2, 3, 4: Tables S2-S4). Peptide coverage was significantly higher for pathogenic strains (26.9% for 7448, and 26.3% for 7422) in comparison to that of the non-pathogenic strain (6.5%). This is evidence not only of a higher P97 expression level in pathogenic strains, but also of a more diverse repertoire of P97 derived segments in the surface of these strains, which would possibly render them more efficient for adhesion.

To further correlate the proteomic profile of each strain to possible functional/physiological features, the identified proteins of the non-pathogenic and pathogenic *M. hyopneumoniae *strains were categorised into COG classes and comparatively analysed. According to the COG functional classification (Additional File 7: Figure S3), most of the identified proteins in the pathogenic 7448 and 7422 strains were assigned to the poorly characterised proteins (Pc) major class, which includes P97, another 18 proteins possibly involved in cytoadherence, and 30 hypothetical proteins.

In 8 COG functional classes, namely D (Cell division and chromosome partitioning), F (Nucleotide transport and metabolism), K (Transcription), L (DNA replication, recombination and repair), M (Cell wall/membrane biogenesis), P (Inorganic ion transport and metabolism), Q (secondary metabolites biosynthesis, transport, and catabolism), and R (General function prediction only), more proteins were detected in the pathogenic strains than in the non-pathogenic one. In the other 5 COG functional classes, namely C (Energy production and conversion), G (Energy production and conversion), J (Translation, ribosomal structure, and biogenesis), O (Post-translational modification, protein turnover, and chaperones), and S (Function unknown), more proteins were detected in the non-pathogenic J strain, with Isp (information storage and processing) being the most prominent major class with 48 identified proteins, including 22 ribosomal proteins.

### Comparative analysis of P97 post-translational modifications

**Figure 3:**
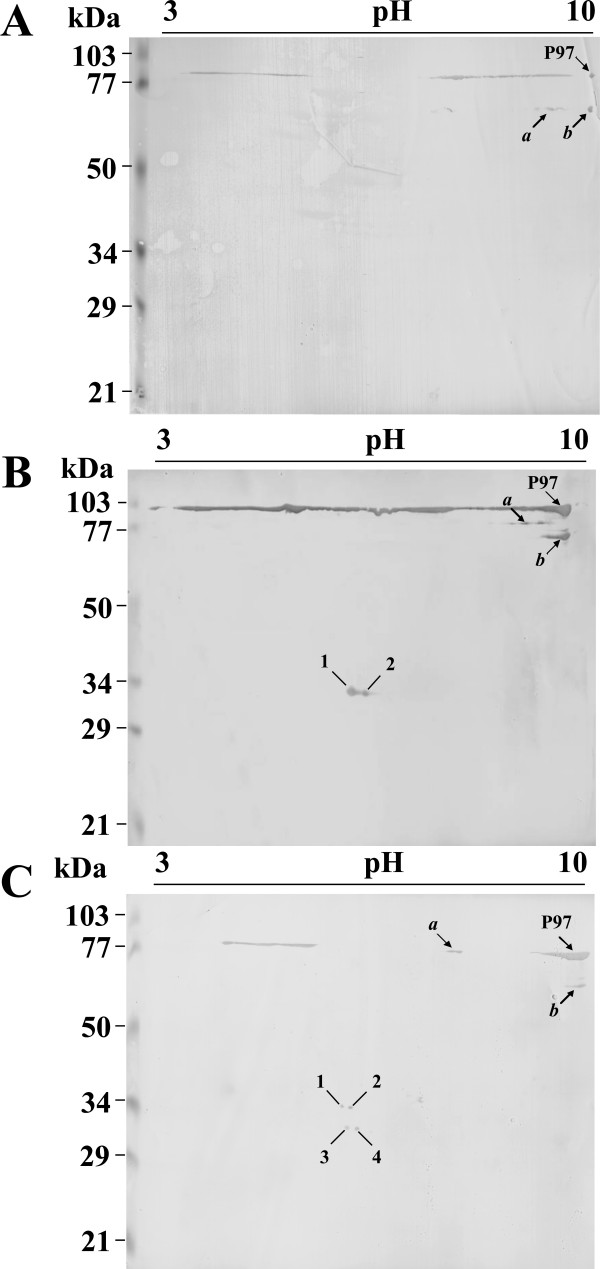
**2DE immunoblotting analyses of P97 adhesin**. Proteins samples from the *M. hyopneumoniae *strains J (C), 7448 (B), and 7422 (A) were resolved by 2DE as described in Figures 1 and 2, electroblotted onto PVDF membranes, and probed with the anti-P97 monoclonal antibody F1B6 (1:400 dilution). Anti-mouse IgG alkaline phosphatase-labelled secondary antibody (1:2000 dilution) was used to develop antigen-antibody reactions. The approximate molecular weights are shown on the left of the gel and the acid-to-alkaline gradient is from left to right. Mature P97 (*P97*), its two main proteolytic products (*a *and *b*), and the P97 pathogenic-strain-specific, low-MW proteolytic products (*1-4*) are indicated.

As different pI P97 isoforms were detected by 2DE and shotgun proteomics in *M. hyopneumoniae *strains J, 7448, and 7422, we further investigated the P97 isoform profiles of each strain by 2DE immunoblotting using a monoclonal antibody directed against the P97 R1 region. As shown in Figure 3A-C, three main P97 isoforms were identified in all strains. One isoform corresponds to the mature P97 adhesin (a polypeptide with 94,412 kDa, pI 9.02, indicated as P97 in the Figure 3) and the other two isoforms (indicated as a and b in the Figure 3) with different MW (between 60 and 90 kDa) and/or pI (between 7.1 and 9.8). In the 7448 and 7422 pathogenic strains, two and four additional low MW species were detected, respectively, without any correspondence to the J non-pathogenic strain.

The two additional P97 isoforms found in the 7448 strain (Figure 3B, spots 1 and 2) presented the same theoretical MW (33 kDa) and pIs of 5.6 and 5.7, respectively. The four additional P97 isoforms of the 7422 strain (Figure 3C, spots 1-4) presented pIs of 5.4 (spots 1 and 3) and 5.6 (spots 2 and 4) and MWs of 34 kDa (spots 1 and 2) and 30 kDa (spots 3 and 4). The P97-derived low pI and low MW spots detected in the 7448 and 7422 strains were consistently observed in the corresponding biological and technical replicates of each strain. The pIs of P97 isoforms estimated based on 2DE did not match to predicted pIs for any of the possible R1-containing, P97-derived peptides with corresponding MW (which were predicted in silico, data not shown), suggesting that the post-translational processing of P97 includes not only proteolytic cleavage, but also pI-changing amino-acid modifications.

## Discussion

We performed a comprehensive and comparative proteomic survey of three strains of the swine pathogen *M. hyopneumoniae*, which was the first study of this scope for a species of this genus of genome-reduced bacteria. Overall, our comparative analyses, including 2DE, immunoblotting, and LC-MS/MS experiments, are suggestive of differential gene expression between *M. hyopneumoniae *strains under culture conditions, which in part is likely related to virulence.

The high degree of similarity in gene content among the genomes of different *M. hyopneumoniae *strains, which have more than 84% of their genes in common [2], suggests that many of the observed physiological differences between strains, including their virulence, are due to differential gene expression. Indeed, in our 2DE analysis, we were able to identify differences in the expression level and the pI and/or MW of several proteins, including surface antigenic proteins related to *M. hyopneumoniae *pathogenicity.

Our LC-MS/MS approach, complementary to the 2DE and immunoblot analyses, resulted in the identification of 35% of the predicted *M. hyopneumoniae *genome protein products. Such coverage is comparable to other recent bacterial proteomic studies, like those of *Halobacterium salinarum *[21,22], *Natronomonas pharaonis *[23], and *Haloferax volcanii *[24], which ranged from 29 to 33%. Since neither a detergent nor another solubilisation agent was used, the analysed *M. hyopneumoniae *samples consist mostly of cytosolic components. Therefore, complementary proteome data is expected to be obtained from future analyses of samples enriched in secreted or membrane-bound proteins.

Differential proteomic studies employing label-free quantification rely on the comparison of peptide abundance as a measure of the corresponding protein level in multiple LC-MS/MS analyses [24]. The emPAI values of proteins in one sample can be compared to those in another sample, and outliers from the emPAI correlation between the two samples can be designated as over- or under-expressed proteins [25]. Although the emPAI may not define the absolute abundance of different proteins, this parameter is useful in estimating the relative abundance of a given protein in different mixtures [25-27], which were samples representative of different *M. hyopneumoniae *strains. A further validation of the emPAI comparative analyses was their consistent concordance with several cases of differential protein expression demonstrated by 2DE.

The comparative emPAI analysis resulted in evidence of pathogenic-strain-specific overexpression of several protein groups. For instance, heat-shock proteins and proteins involved in pyruvate metabolising pathways, such as acetate kinase, pyruvate dehydrogenase E1-alpha subunit, pyruvate dehydrogenase E1-beta subunit, pyruvate kinase, and lactate dehydrogenase, were shown to be overexpressed in the 7448 and 7422 strains. These results are consistent with the idea that a functional heat shock response, already detected in *M. hyopneumoniae *and other related species, is important for responding to temperature stress during host pyrexia [11,28,29]. Additionally, the capacity to use glucose and other alternative carbon sources, such as glycerol and fructose also present in *M. pneumoniae *and other *Mycoplasma *species [30,31], would be important for *M. hyopneumoniae *to cope with predicted changes in the availability of carbon sources in its habitat.

Another important group of proteins with differential expression between strains is the redox balancing proteins. The response of the swine immune system to the presence of *M. hyopneumoniae *in the consequent inflammatory process that includes the production of superoxides [32] and a mechanism to evade that response would be quite adaptive for the pathogen. In agreement with this hypothesis, most of the differentially expressed oxidative stress response proteins were significantly more represented in the pathogenic strains. *M. hyopneumoniae *lacks antioxidants such as catalase and superoxide dismutase [2], but, from a repertoire of five detected oxidative stress response proteins, four proteins (i.e., thioredoxin, NADH oxidase, methionine sulfoxide reductase, and thioredoxin reductase) were differentially expressed and over-represented in samples from at least one of the pathogenic strains in comparison to the J strain.

Antigenic surface proteins constituted a well-represented group among the LC MS/MS identified proteins with 13 members, including P97 and its two P97-like orthologs as well as the two P102 orthologs [2]. P97, P102, and at least some of the other identified antigenic surface proteins are involved in the adherence of *M. hyopneumoniae *to the mucosa of the distal portion of the respiratory tract of swine, which is a fundamental step in the development of EP [7,33]. Our samples were from in vitro cultures, which did not have any contact with host tissue, and the large representation of surface adhesion-related proteins could be a result of induction of their expression by Friis medium components, such as proteins and signalling molecules from pig serum. An alternative explanation would be a somewhat "constitutive" mode of expression of the repertoire of adhesins, which would render the bacterium competent for infection and adhesion prior to direct interaction with the swine respiratory epithelium.

In the comparative emPAI analysis, however, of all the identified surface and/or adhesion-related proteins, only P97 isoforms showed significant differences in their expression levels and were more prominent in both the pathogenic strains than in the J strain. Therefore, the adhesion-deficient [34] J strain is not significantly devoid of adhesins, but our 2DE and immunoblot results suggest both qualitative and quantitative underrepresentation of at least three important adhesins, namely P97, P146, and P216, in comparison to the 7448 and 7422 strains.

Overall, the non-pathogenic strain protein expression profile is suggestive of a non-infective proliferate lifestyle with most of the identified proteins assigned to the COG Isp major class, which is comprised of the translation, ribosomal structure, and biogenesis category; the transcription category; and the DNA replication, recombination, and repair category. For the pathogenic 7448 and 7422 strains, most of the identified proteins were assigned to the poorly characterised proteins (Pc) major class, which is comprised of proteins with only general function prediction and unknown function, including proteins known to be involved in cytoadherence, and several hypothetical proteins, at least six of which were predicted as surface proteins and four of which were predicted as secreted proteins (our unpublished data). Distribution of the expressed proteins in the avirulent versus virulent strains suggests that the differential presence/absence or abundance of expressed proteins may be related to their ability to infect a suitable swine host, provided that experimental support to the previous assumptions is based on genomic analyses [2].

The described repertoire of *M. hyopneumoniae *differentially expressed proteins showed little correlation (data not shown) with previous *M. hyopneumoniae *232 transcriptomic analysis [11-15]. This may, at least in part, due the use of different strains or different assay conditions. However, it is reasonable to assume, considering that *M. hyopneumoniae *genome is poor in genes coding for known transcriptional regulators [2], that regulation processes at the translational and post-translational levels are relevant for this species. Importantly, the main adhesin P97 presented no significative expression difference at the transcriptional level in all different conditions so far analysed [11-15], while, in our proteomic analyses, it presented both quantitative and qualitative interstrain differences.

The P97 adhesin is considered an important virulence factor for *M. hyopneumoniae *and variation in the number of tandem amino acid repeats in the R1 region of this protein has been correlated to the bacterial capacity of adhesion [35]. The 121 kDa P97 precursor is generated by peptide-signal cleavage, possibly in concert with P97 translocation to the membrane [36]. It has also been demonstrated that mature P97 is further proteolytically processed to generate smaller derived polypeptides containing the R1 repeat [8]. Further extending these previous observations regarding P97 post-translational processing, we were able to demonstrate differences in P97 proteolytic cleavage between strains, generating different numbers of R1-containing polypeptides in the pathogenic 7448 and 7422 strains that were not observed in the non-pathogenic J strain. The P97-derived low MW polypeptides detected by 2DE immunoblotting in the pathogenic strains presented pIs lower than those predicted for any theoretical P97-derived polypeptides including the R1 repeat, which is evidence that these 30-34 kDa P97 proteolytic products also undergo some sort of post-translational pI-decreasing amino acid modification (e.g., acetylation, carbamylation, or phosphorylation). Initially, it was assumed that, as a classical transmembrane protein, P97 would be translocated to the cell surface through the general secretory pathway. There it would remain attached to the cell membrane by its transmembrane domain and would expose its cilium-binding motif to the extracellular milieu. However, previous evidence that P97 proteolytic cleavage may separate its transmembrane and cilium-binding domains [36,37] and that different protein fragments including the R1-containing cilium binding motif are also generated [8] already pointed to an alternative mechanism for regulating the exposure of the P97 cilium-binding domain in the *M. hyopneumoniae *cell surface. Our results add further complexity to this scenario, as they show that the repertoire of proteolytic P97 peptide, including the adhesion-related R1 repeat, vary between strains with different virulence properties. Therefore, further experiments will be necessary to investigate which P97 fragments are actually presented on the cell surface, how they reach and stay in that site, and what the actual contribution of each of them is for in the adhesion of *M. hyopneumoniae *to the swine respiratory epithelium.

Besides P97, there are other high-MW *M. hyopneumoniae *proteins which undergo proteolytic processing [16,38,39], suggesting that this is an important post-translational process in the physiology of this species. Proteolytic processing has not been described for any other *Mycoplasma *species (or at least not to this extent), although the protease repertoires of these species do not differ significantly from that found in the *M. hyopneumoniae *genome [2,3]. Of the total of 13 proteases found in the *M. hyopneumoniae *genome [40], 8 had their expression confirmed by our shotgun proteomic analysis, but again, this did not significantly different from the repertoire of proteases expressed in other mycoplasmas [41-44]. Therefore, assuming a common repertoire and expression pattern of proteases in different *Mycoplasma *species, the apparent larger number of proteins post-translationally processed by proteolysis in *M. hyopneumoniae *may be evidence of particular regulatory mechanisms and/or recent mutations leading to susceptibility to proteolytic processing in some key target proteins.

## Conclusions

In conclusion, approximately one-third of the total proteome of *M. hyopneumoniae *was identified in a comprehensive inter-strain analysis, which showed that the repertoires of identified proteins of the two pathogenic strains are as different from each other as they are from the non-pathogenic one. This is indicative of significant inter-strain variability for this species not necessarily related to pathogenicity, although some potentially virulence-related differences in protein expression have been detected. Our results improve the current annotation of *M. hyopneumoniae *genome by a careful analysis of high-scoring proteins coded by genes previously regarded as hypothetical, thereby providing further insights into *M. hyopneumoniae *biology. The current characterised fraction of the *M. hyopneumoniae *proteome and its future expansion through proteomic analyses of changing growth conditions and various stress challenges will serve as a useful research resource, complementary to the increasing amount of data from comparative genomic and transcriptomic analyses and subsidiary to functional studies of specific genes and proteins.

## Methods

### Bacterial strains, cultivation, and cell protein extracts

*M. hyopneumoniae *strain J (ATCC 25934), a nonpathogenic strain with reduced adhesion capacity to porcine cilia, was acquired from American Type Culture Collection by the Empresa Brasileira de Pesquisa Agropecuária-Centro Nacional de Pesquisa de Suínos e Aves (EMBRAPA-CNPSA, Concórdia, Santa Catarina, Brazil). *M. hyopneumoniae *pathogenic strain 7448 was isolated from infected swine from Lindóia do Sul (Santa Catarina, Brazil) [2]. *M. hyopneumoniae *pathogenic strain 7422, a field isolate from Concórdia (Santa Catarina, Brazil), was obtained from the EMBRAPA-CNPSA collection. Isolation and cultivation were performed under standard conditions, as described by Friis (1975) [45], with cells grown in 2 L of medium until they reached a density of 10^8 ^CFU mL^-1^.

For protein extract preparation, cells were harvested by centrifugation at 18,000 × g for 10 min and resuspended in 1 mL of 25 mM Tris-HCl, pH 7.2. Cell suspensions were then lysed by sonication at 25 Hz in an ice bath by five 30 s cycles with a 1 min interval between pulses. Proteins were quantified using the Bradford method (Bio-Rad Protein Assay, Bio-Rad). For each strain, protein extracts were prepared from three identical and independent cultures (biological replicates) and then mixed into a single protein sample.

### Two-dimensional gel electrophoresis and gel image analysis

Protein samples were solubilised in isoelectric focusing (IEF) buffer containing 7 M urea, 2 M thio-urea, 4% (w/v) CHAPS, 1% (w/v) dithiothreitol (DTT), and 0.2% (v/v) ampholytes pH 3-10 (Bio-Rad, Hercules, US). The 17 cm immobilised pH gradient (IPG) strips (pH 3-10 or 4-7, Bio-Rad, Hercules, US) were passively rehydrated for 16 h with 300 μL of cell extract samples containing 1-2 mg of protein. IEF was performed in a Protean IEF cell system (Bio-Rad, Hercules, US) with up to 50,000 VH at a maximum voltage of 10,000 V. Strips were equilibrated for 15 min in equilibration buffer I (30%, v/v, glycerol, 6 M urea, 1% DTT, a trace of bromophenol blue) and for 15 min in equilibration buffer II (equilibration solution I with DTT replaced by 4% iodoacetamide). In the second dimension, IPG strips were run vertically onto SDS-PAGE 12% gels using PROTEAN^® ^II xi 2D Cell (Bio-Rad, Hercules, US). For each protein sample, three independent gels were run (technical replicates). Gels were stained with 0.1% Coomassie Brilliant Blue G (Acros, Geel, Belgium) and scanned with a computer-assisted G-800 densitometer (Bio-Rad, Hercules, US). The 2DE image analyses were carried out using the PDQuest 8.0 software package [46]. After background subtraction, spot detection and match, one standard gel, representative of a given strain sample, was defined. Spots in the standard gels were then matched to the previously reported *M. hyopneumoniae *7448 proteomic maps [16] for protein identification, and proteins differentially expressed between strains were identified by comparison between gels, also performed using the PDQuest 8.0 software.

### Liquid chromatography tandem mass spectrometry

To identify proteins, we used a liquid chromatography (LC) separation (reversed-phase HPLC) coupled with tandem mass spectrometry (MS/MS) strategy. MS/MS analyses were performed in an electrospray ionisation (ESI) quadrupole time-of-flight (Q-TOF) Ultima API mass spectrometer (Micromass, Manchester, UK) coupled to a capillary liquid chromatography system (CapLC, Waters, Milford, US). A nanoflow ESI source was used with a lockspray source for lockmass measurement during all the chromatographic runs. Samples of approximately10 mg of each *M. hyopneumoniae *strain protein extract were digested with trypsin (Promega, Madison, US) and the resulting peptide mixture was desalted using an OASIS^® ^HLB Cartridge column (Waters, Milford, US). The peptides were separated in a Nanoease C18 (75 μm ID) capillary column by elution with a water/acetonitrile 0.1% formic acid gradient. Data were acquired in data-dependent mode (DDA), and multiple charged peptide ions (+2 and +3) were automatically mass selected and dissociated in MS/MS experiments. Typical LC and ESI conditions were flow of 200 nL/min, nanoflow capillary voltage of 3.5 kV, block temperature of 100°C, and cone voltage of 100 V. For each protein sample, three independent LC-MS/MS were performed.

### Data processing and bioinformatics analyses

The MS/MS spectra were processed using Proteinlynx v. 2.0 software (Waters, Milford, US) and the generated PKL files were used to perform database searches using the MASCOT software v. 2.2 (Matrix Science, London, UK) against the non-redundant NCBI database (9,251,875 sequences and 3,169,794,832 residues, at Jul 7, 2009). Search parameters allowed a maximum of one missed cleavage, the carbamidomethylation of cysteine, the possible oxidation of methionine, peptide tolerance of 0.2 Da, and MS/MS tolerance of 0.1 Da. The significance threshold was set at p < 0.05, and identification required that each protein contained at least one peptide with an expected value < 0.05.

To gauge the false positive peptide matching rate in our analysis, we applied the target-decoy search strategy by searching the MS/MS spectra against the reversed and randomised *M. hyopneumoniae *proteome sequences [47].

For each protein match identified by MASCOT, the software calculated the corresponding exponentially modified protein abundance index (emPAI) [25] as the transformed ratio of the number of experimentally observed peptides to the total number of peptides that can theoretically be detected within the operating mass range and retention range of the instrument. In the comparisons of emPAI values of a given protein between strains, differences were considered significant when there was at least a twofold difference between the calculated emPAIs [25]. Since emPAI is a logarithmic index, significant differences were obtained only when protein abundances were calculated based on the detection of more than a single peptide per protein per sample, avoiding consideration of cases of proteins identified by unique peptides.

The Clusters of Orthologous Groups (COG) classification of each of the identified proteins was obtained from the Southern Genome Investigation Program *M. hyopneumoniae *Genome Database http://www.genesul.lncc.br.

The Compute pI/Mw tool from the ExPASy Proteomics Server http://ca.expasy.org/ was used to estimate pI and MW corresponding to proteins/polypeptides represented in 2DE spots.

### Immunoblotting

Proteins were resolved by 2DE and electroblotted onto PVDF membranes (GE Healthcare, Chalfont St. Giles, UK). Blotted membranes were blocked with 5% (w/v) skim milk powder in PBS (10 mM Na_2_HPO_4_, 1.7 mM KH_2_PO_4_, 137 mM NaCl, 2.7 mM KCl) and then incubated with an anti-P97 monoclonal antibody (F1B6, purchased from Iowa University) [36] diluted 1:400 in blocking solution. Membranes were washed three times in PBS for 10 min, incubated with a secondary antibody (anti-mouse IgG alkaline phosphatase conjugate, Sigma-Aldrich, St. Louis, US) diluted 1:2000, washed, and developed with NBT/BCIP (Sigma-Aldrich, St. Louis, US). For each protein sample, three independent immunoblot experiments were performed (technical replicates).

## Competing interests

The authors declare that they have no competing interests.

## Authors' contributions

PMP carried out sample preparation, and 2DE, immunobloting, mass spectrometry and bioinformatic analyses; CSK carried out *M. hyopneumoniae *cultivation; HBF and AZ conceived the study, and participated in its design, implementation, and coordination. All authors read and approved the final manuscript.

## Supplementary Material

Additional file 1 **Table S1 - The emPAI value comparison and differentially expressed proteins**. Click here for file

Additional file 2 **Table S2 - Identification of J strain proteins by LC-MS/MS**. Click here for file

Additional file 3 **Table S3 - Identification of 7448 strain proteins by LC-MS/MS**. Click here for file

Additional file 4 **Table S4 - Identification of 7422 strain proteins by LC-MS/MS**. Click here for file

Additional file 5 **Figure S1 - Venn diagram of the protein sets**. Venn diagram obtained from the comparison of LC-MS/MS identified protein repertoires of *M. hyopneumoniae *strains J, 7448, and 7422. Click here for file

Additional file 6 **Figure S2 - Comparative analyses of *M. hyopneumoniae *strains J, 7448, and 7422 LC-MS/MS proteomes based on emPAI relative abundance**. (A-C) Bivariant plots of emPAI values (average of three independent LC-MS/MS analysis, with less than 5% difference between than) of proteins from: strain J against strain 7448 (A); strain J against strain 7422 (B); and strain 7448 against strain 7422 (C). Plotted protein emPAI values are in Table S1 (Additional File). Proteins outside the V-shaped shaded area (marked in gray) were assumed to be overexpressed in the strain whose emPAI values are assigned to the proximal axis. For overexpression assumption, a two-fold or higher difference between the emPAI values for a given protein in the two compared strains was required. Click here for file

Additional file 7 **Figure S3 - COG functional classification of identified proteins from *M. hyopneumoniae *strains J, 7448, and 7422**. Schematic representations of identified proteins belonging to the COG functional classes as follows: Major class Information storage and processes: (J) Translation, ribosomal structure and biogenesis, (K) Transcription, (L) DNA replication, recombination, and repair; Major class Cellular processes: (D) Cell division and chromosome partitioning, (O) Post-translational modification, protein turnover, and chaperones, (M) Cell envelope biogenesis, outer membrane, (N) Cell motility and secretion, (P) Inorganic ion transport and metabolism; Major class Metabolism: (C) Energy production and conversion, (G) Carbohydrate transport and metabolism, (E) Amino acid transport and metabolism, (F) Nucleotide transport and metabolism, (H) Coenzyme metabolism, (I) Lipid metabolism, (Q) Secondary metabolites biosynthesis, transport, and catabolism; Major class Poorly characterised: (R) General function prediction only, (S) Function unknown. Click here for file
