# Supplementary material for: Comparative proteomic analysis of pathogenic and non-pathogenic strains from the swine pathogen Mycoplasma hyopneumoniae
Source: Proteome Sci. 2009 Dec 21;7:45. doi: 10.1186/1477-5956-7-45 (PMC2804596; doi:10.1186/1477-5956-7-45)
Supplement: Additional file 1 — Table S1 - The emPAI value comparison and differentially expressed proteins. [file 1477-5956-7-45-S1.PDF]

Table 1

Identification of J strain proteins by LC-MS/MS. Protein identified by a liquid chromatography (LC) separation (reversed-phase HPLC) coupled with a tandem mass spectrometry (MS/MS) by searching *M. hyopneumoniae* strain J protein databases using MASCOT search engine.

Accession number[1]	Protein description[2]	MASCOT score[3]	Sequence coverage (%)	COG[4]
gi|71893362|ref|YP_278808.1|	glucose-inhibited division protein A	16	3.3	D
gi|71893368|ref|YP_278814.1|	hypothetical protein MHJ_0009	252	10.8	S
gi|71893370|ref|YP_278816.1|	heat shock protein	172	20.0	O
gi|71893373|ref|YP_278819.1|	fructose-bisphosphate aldolase	288	49.1	G
gi|71893387|ref|YP_278833.1|	isoleucyl-tRNA synthetase	78	12.2	J
gi|71893390|ref|YP_278836.1|	glyceraldehyde 3-phosphate dehydrogenase	476	37.8	G
gi|71893407|ref|YP_278853.1|	ATP synthase gamma chain	23	6.0	C
gi|71893408|ref|YP_278854.1|	ATP synthase subunit B	230	30.1	C
gi|71893411|ref|YP_278857.1|	elongation factor Ts	79	22.1	J
gi|71893422|ref|YP_278868.1|	molecular chaperone DnaK	1508	38.5	O
gi|71893426|ref|YP_278872.1|	bacterial nucleoid DNA-binding protein	272	43.8	L
gi|71893430|ref|YP_278876.1|	elongation factor EF-2	161	19.1	K
gi|71893431|ref|YP_278877.1|	30S ribosomal protein S7	46	24.4	J
gi|71893437|ref|YP_278883.1|	NADH oxidase	940	35.2	R
gi|71893438|ref|YP_278884.1|	thymidine phosphorylase	85	24.4	F
gi|71893439|ref|YP_278885.1|	purine-nucleoside phosphorylase	239	22.0	F

gi|71893450|ref|YP_278896.1|	hypothetical protein MHJ_0091	42	17.0	S
gi|71893452|ref|YP_278898.1|	thiol peroxidase	242	38.4	O
gi|71893454|ref|YP_278900.1|	thioredoxin reductase	367	27.5	O
gi|71893457|ref|YP_278903.1|	ATP-dependent protease binding protein	144	16.6	O
gi|71893458|ref|YP_278904.1|	triosephosphate isomerase	13	9.0	G
gi|71893462|ref|YP_278908.1|	P97 paralog 1	258	18.8	S
gi|71893463|ref|YP_278909.1|	DNA gyrase subunit B	166	16.3	L
gi|71893464|ref|YP_278910.1|	6-phosphofructokinase	101	20.2	G
gi|71893467|ref|YP_278913.1|	adenine phosphoribosyltransferase	861	52.1	F
gi|71893468|ref|YP_278914.1|	pyruvate dehydrogenase E1-alpha subunit	1149	41.2	C
gi|71893469|ref|YP_278915.1|	pyruvate dehydrogenase	1731	55.4	C
gi|71893472|ref|YP_278918.1|	hypothetical protein MHJ_0115	29	35.4	S
gi|71893476|ref|YP_278922.1|	50S ribosomal protein L20	30	25.6	J
gi|71893479|ref|YP_278925.1|	pyruvate kinase	102	26.7	G
gi|71893482|ref|YP_278928.1|	aminopeptidase	48	4.0	G
gi|71893484|ref|YP_278930.1|	50S ribosomal protein L21	138	17.0	J
gi|71893485|ref|YP_278931.1|	50S ribosomal protein L27	29	36.9	J
gi|71893486|ref|YP_278932.1|	lipase-esterase	86	13.2	R
gi|71893489|ref|YP_278935.1|	L-lactate dehydrogenase	290	45.1	C
gi|71893490|ref|YP_278936.1|	hypothetical protein MHJ_0134	56	3.0	S

gi|71893491|ref|YP_278937.1|	peptide chain release factor 1	68	7.0	J
gi|71893500|ref|YP_278946.1|	hypothetical protein MHJ_0144	73	23.8	S
gi|71893501|ref|YP_278947.1|	trigger factor	312	27.7	O
gi|71893513|ref|YP_278959.1|	phosphopentomutase	48	3.0	G
gi|71893521|ref|YP_278967.1|	30S ribosomal protein S11	70	34.3	J
gi|71893522|ref|YP_278968.1|	30S ribosomal protein S13	99	15.3	J
gi|71893528|ref|YP_278974.1|	50S ribosomal protein L15	42	17.2	J
gi|71893531|ref|YP_278977.1|	50S ribosomal protein L6	284	33.5	J
gi|71893532|ref|YP_278978.1|	30S ribosomal protein S8	83	16.0	J
gi|71893534|ref|YP_278980.1|	50S ribosomal protein L5	130	23.3	J
gi|71893535|ref|YP_278981.1|	50S ribosomal protein L24	87	15.0	J
gi|71893541|ref|YP_278987.1|	50S ribosomal protein L22	58	22.4	J
gi|71893542|ref|YP_278988.1|	30S ribosomal protein S19	52	28.9	J
gi|71893543|ref|YP_278989.1|	50S ribosomal protein L2	248	33.0	J
gi|71893545|ref|YP_278991.1|	50S ribosomal protein L4	74	18.0	J
gi|71893546|ref|YP_278992.1|	50S ribosomal protein L3	58	18.7	J
gi|71893550|ref|YP_278996.1|	protein P97	1638	6.5	S
gi|71893551|ref|YP_278997.1|	protein P102	693	15.5	S
gi|71893558|ref|YP_279004.1|	cell division protein	149	24.6	O
gi|71893559|ref|YP_279005.1|	lysyl-tRNA synthetase	109	8.0	J

gi|71893560|ref|YP_279006.1|	hydrolase of the HAD family	28	12.0	R
gi|71893564|ref|YP_279010.1|	oligopeptide ABC transporter system permease	127	16.9	E
gi|71893566|ref|YP_279012.1|	oligopeptide ABC transporter ATP-binding protein	55	16.7	E
gi|71893567|ref|YP_279013.1|	oligopeptide ABC transporter ATP-binding protein	39	18.6	E
gi|71893568|ref|YP_279014.1|	hypothetical protein MHJ_0212	424	19.0	S
gi|71893569|ref|YP_279015.1|	lipoprotein	106	14.6	S
gi|71893571|ref|YP_279017.1|	ribonucleotide-diphosphate reductase alpha subunit	160	21.5	F
gi|71893572|ref|YP_279018.1|	hypothetical protein MHJ_0216	106	36.7	S
gi|71893573|ref|YP_279019.1|	ribonucleotide-diphosphate reductase beta subunit	64	34.0	F
gi|71893574|ref|YP_279020.1|	serine hydroxymethyltransferase	14	8.0	E
gi|71893575|ref|YP_279021.1|	methylmalonate-semialdehyde dehydrogenase	86	22.7	C
gi|71893579|ref|YP_279025.1|	myo-inositol catabolism protein	25	16.0	G
gi|71893589|ref|YP_279035.1|	protein-export membrane protein	29	12.0	N
gi|71893592|ref|YP_279038.1|	hypothetical protein MHJ_0236	45	25.0	S
gi|71893595|ref|YP_279041.1|	TRSE-like protein	23	2.0	N
gi|71893598|ref|YP_279044.1|	phosphopyruvate hydratase	313	37.9	G
gi|71893599|ref|YP_279045.1|	seryl-tRNA synthetase	39	21.0	J
gi|71893600|ref|YP_279046.1|	hypothetical protein MHJ_0244	62	33.0	S
gi|71893601|ref|YP_279047.1|	triacylglycerol lipase	26	23.0	R
gi|71893602|ref|YP_279048.1|	lipoate-protein ligase A	100	17.7	H

gi|71893604|ref|YP_279050.1|	DNA polymerase III subunits gamma and tau	14	5.0	L
gi|71893605|ref|YP_279051.1|	hypothetical protein MHJ_0249	56	27.0	S
gi|71893610|ref|YP_279056.1|	hypoxanthine-guanine phosphoribosyltransferase	132	21.9	F
gi|71893614|ref|YP_279060.1|	DNA ligase	13	5.0	L
gi|71893620|ref|YP_279066.1|	P97 paralog 2	36	16.0	S
gi|71893621|ref|YP_279067.1|	phenylalanyl-tRNA synthetase alpha chain	26	15.0	J
gi|71893622|ref|YP_279068.1|	phenylalanyl-tRNA synthetase beta subunit	152	23.1	J
gi|71893628|ref|YP_279074.1|	CTP synthetase	49	17.2	F
gi|71893639|ref|YP_279085.1|	hypothetical protein MHJ_0283	65	23.1	S
gi|71893643|ref|YP_279089.1|	30S ribosomal protein S6	148	19.1	J
gi|71893644|ref|YP_279090.1|	hypothetical protein MHJ_0288	95	20.5	S
gi|71893650|ref|YP_279096.1|	permease	15	2.0	G
gi|71893700|ref|YP_279146.1|	hypothetical protein MHJ_0347	26	21.0	S
gi|71893701|ref|YP_279147.1|	P60-like lipoprotein	42	8.1	S
gi|71893706|ref|YP_279152.1|	amino acid permease	20	4.0	E
gi|71893715|ref|YP_279161.1|	lipoprotein	168	11.4	S
gi|71893721|ref|YP_279167.1|	Lppt protein	628	24.0	S
gi|71893722|ref|YP_279168.1|	hypothetical protein MHJ_0369	452	22.6	S
gi|71893724|ref|YP_279170.1|	PTS system enzyme IIB component	201	14.0	G
gi|71893726|ref|YP_279172.1|	hypothetical protein MHJ_0373	1042	43.1	S

gi|71893727|ref|YP_279173.1|	lipoprotein	494	24.3	S
gi|71893733|ref|YP_279179.1|	thioredoxin	174	30.9	O
gi|71893755|ref|YP_279201.1|	hypothetical protein MHJ_0404	18	6.0	S
gi|71893760|ref|YP_279206.1|	methionine--tRNA ligase	40	14.0	J
gi|71893765|ref|YP_279211.1|	asparaginyl-tRNA synthetase	99	10.7	J
gi|71893767|ref|YP_279213.1|	ATP-dependent helicase PcrA	177	19.5	L
gi|71893768|ref|YP_279214.1|	hypothetical protein MHJ_0417	67	15.0	S
gi|71893769|ref|YP_279215.1|	Holliday junction DNA helicase motor protein	19	4.0	L
gi|71893777|ref|YP_279223.1|	transketolase	288	28.0	G
gi|71893779|ref|YP_279225.1|	tRNA (5-methylaminomethyl-2-thiouridylate)-methyltransferase	19	7.3	J
gi|71893787|ref|YP_279233.1|	3-hexulose-6-phosphate synthase	172	22.1	G
gi|71893793|ref|YP_279239.1|	hypothetical protein MHJ_0442	16	3.0	S
gi|71893796|ref|YP_279242.1|	hypothetical protein MHJ_0445	175	15.6	S
gi|71893803|ref|YP_279249.1|	acyl carrier protein phosphodiesterase	73	14.9	I
gi|71893807|ref|YP_279253.1|	50S ribosomal protein L1	197	45.9	J
gi|71893808|ref|YP_279254.1|	50S ribosomal protein L11	128	32.9	J
gi|71893812|ref|YP_279258.1|	leucyl aminopeptidase	128	32.7	E
gi|71893814|ref|YP_279260.1|	hypothetical protein MHJ_0463	127	15.7	S
gi|71893816|ref|YP_279262.1|	hypothetical protein MHJ_0465	32	15.4	S
gi|71893820|ref|YP_279266.1|	phosphoenolpyruvate-protein phosphotransferase	248	36.7	G

gi|71893826|ref|YP_279272.1|	ATP synthase subunit B	127	18.3	C
gi|71893827|ref|YP_279273.1|	ATP synthase subunit A	46	2.9	C
gi|71893830|ref|YP_279276.1|	hypothetical protein MHJ_0479	31	9.7	S
gi|71893831|ref|YP_279277.1|	hypothetical protein MHJ_0480	85	12.2	S
gi|71893837|ref|YP_279283.1|	hypothetical protein MHJ_0486	78	13.0	S
gi|71893838|ref|YP_279284.1|	phosphoglycerate kinase	252	22.3	G
gi|71893842|ref|YP_279288.1|	mannose-6-phosphate isomerase	154	40.4	G
gi|71893844|ref|YP_279290.1|	P216 surface protein	2746	46.5	S
gi|71893845|ref|YP_279291.1|	P76 membrane protein precursor	1931	30.4	S
gi|71893854|ref|YP_279300.1|	dihydrolipoamide acetyltransferase	269	23.5	C
gi|71893855|ref|YP_279301.1|	dihydrolipoamide dehydrogenase	271	10.2	C
gi|71893856|ref|YP_279302.1|	acetate kinase	290	26.4	C
gi|71893857|ref|YP_279303.1|	phosphate acetyltransferase	316	28.7	C
gi|71893862|ref|YP_279308.1|	46K surface antigen precursor	514	42.0	S
gi|71893873|ref|YP_279319.1|	oligoendopeptidase F	122	18.1	E
gi|71893875|ref|YP_279321.1|	elongation factor Tu	1178	55.2	J
gi|71893876|ref|YP_279322.1|	heat shock ATP-dependent protease	437	22.4	O
gi|71893879|ref|YP_279325.1|	deoxyribose-phosphate aldolase	219	33.0	F
gi|71893880|ref|YP_279326.1|	DNA gyrase subunit A	125	20.5	L
gi|71893883|ref|YP_279329.1|	glucose-6-phosphate isomerase	96	5.8	G

gi|71893886|ref|YP_279332.1|	ribosome recycling factor	34	9.8	J
gi|71893908|ref|YP_279354.1|	PTS system galactitol-specific enzyme IIB component	28	13.2	G
gi|71893920|ref|YP_279366.1|	hypothetical protein MHJ_0571	17	1.9	S
gi|71893921|ref|YP_279367.1|	dihydrolipoamide dehydrogenase	89	23.3	C
gi|71893934|ref|YP_279380.1|	translation initiation factor IF-2	20	4.0	J
gi|71893937|ref|YP_279383.1|	glycerol-3-phosphate dehydrogenase	18	6.8	R
gi|71893941|ref|YP_279387.1|	ATP-binding protein	171	21.9	L
gi|71893944|ref|YP_279390.1|	phosphoglyceromutase	93	22.2	G
gi|71893952|ref|YP_279398.1|	hypothetical protein MHJ_0603	14	5.0	S
gi|71893954|ref|YP_279400.1|	inorganic pyrophosphatase	43	10.8	C
gi|71893955|ref|YP_279401.1|	ABC transporter xylose-binding lipoprotein	575	34.2	R
gi|71893956|ref|YP_279402.1|	sugar ABC transporter ATP-binding protein	88	13.5	R
gi|71893966|ref|YP_279412.1|	DNA-directed RNA polymerase beta' subunit	143	23.3	K
gi|71893968|ref|YP_279414.1|	50S ribosomal protein L7/L12	94	24.0	J
gi|71893971|ref|YP_279417.1|	lipoprotein	51	13.2	S
gi|71893972|ref|YP_279418.1|	DNA adenine methylase	23	2.0	L
gi|71893979|ref|YP_279425.1|	5'-nucleotidase precursor	101	19.7	F
gi|71893984|ref|YP_279430.1|	O-sialoglycoprotein endopeptidase	101	25.2	O
gi|71893985|ref|YP_279431.1|	transcription antitermination protein NusG	25	4.0	K
gi|71893998|ref|YP_279444.1|	30S ribosomal protein S9	26	24.2	J

gi|71893999|ref|YP_279445.1|	50S ribosomal protein L13	30	13.2	J
gi|71894003|ref|YP_279449.1|	glucose-inhibited division protein B	17	12.0	M
gi|71894004|ref|YP_279450.1|	prolipoprotein p65	1431	37.2	S
gi|71894005|ref|YP_279451.1|	ABC transporter ATP-binding protein P115-like	108	20.1	D
gi|71894010|ref|YP_279456.1|	hypothetical protein MHJ_0662	896	20.2	S
gi|71894011|ref|YP_279457.1|	adhesin like-protein P146	1015	21.7	S
gi|71894015|ref|YP_279461.1|	transcription elongation factor	59	32.5	K
gi|71894021|ref|YP_279467.1|	hypothetical protein MHJ_0673	183	10.6	S

[1] CDS access number in the NCBI database (http://www.ncbi.nlm.nih.gov).
[2] Protein identification according to NCBI database (http://www.ncbi.nlm.nih.gov).
[3] MASCOT score is -10 x log(*P*), where *P* is the probability that the observed match is a random event.
[4] COG database functional classes: (J) Translation, ribosomal structure and biogenesis, (K) Transcription, (L) DNA replication, recombination and repair, (D) Cell division and chromosome partitioning, (O) Posttranslational modification, protein turnover, chaperones, (M) Cell envelope biogenesis, outer membrane, (N) Cell motility and secretion, (C) Energy production and conversion, (G) Carbohydrate transport and metabolism, (E) Amino acid transport and metabolism, (F) Nucleotide transport and metabolism, (H) Coenzyme metabolism, (I) Lipid metabolism, transport and catabolism,(R) General function prediction only, and (S) Function unknown.
