# Supplementary material for: Comparative proteomic analysis of pathogenic and non-pathogenic strains from the swine pathogen Mycoplasma hyopneumoniae
Source: Proteome Sci. 2009 Dec 21;7:45. doi: 10.1186/1477-5956-7-45 (PMC2804596; doi:10.1186/1477-5956-7-45)
Supplement: Additional file 2 — Table S2 - Identification of J strain proteins by LC-MS/MS. [file 1477-5956-7-45-S2.PDF]

Table 2

Identification of 7448 strain proteins by LC-MS/MS. Protein identified by a liquid chromatography (LC) separation (reversed-phase HPLC) coupled with a tandem mass spectrometry (MS/MS) by searching *M. hyopneumoniae* strain 7448 protein databases using MASCOT search engine.

Accession number[1]	Protein description[2]	MASCOT score[3]	Sequence coverage (%)	COG[4]
gi|72080345|ref|YP_287403.1|	glucose-inhibited division protein A	15	3.1	D
gi|72080348|ref|YP_287406.1|	DHH family phosphoesterase	32	9.1	R
gi|72080351|ref|YP_287409.1|	hypothetical protein MHP7448_0009	76	18.4	S
gi|72080353|ref|YP_287411.1|	heat shock protein	54	18.4	O
gi|72080356|ref|YP_287414.1|	fructose-bisphosphate aldolase	94	30.7	G
gi|72080361|ref|YP_287419.1|	ABC transporter ATP-binding protein	55	13.3	R
gi|72080365|ref|YP_287423.1|	ABC transporter ATP-binding protein	26	1	R
gi|72080373|ref|YP_287431.1|	lipoprotein signal peptidase	23	10	M
gi|72080374|ref|YP_287432.1|	isoleucyl-tRNA synthetase	39	17.8	J
gi|72080377|ref|YP_287435.1|	glyceraldehyde 3-phosphate dehydrogenase	575	16.1	G
gi|72080379|ref|YP_287437.1|	VACB-like ribonuclease II	14	1	K
gi|72080383|ref|YP_287441.1|	GTP-binding protein Obg	17	4.8	R
gi|72080395|ref|YP_287453.1|	ATP synthase subunit B	94	9.6	C
gi|72080397|ref|YP_287455.1|	30S ribosomal protein S2	173	7.3	J
gi|72080398|ref|YP_287456.1|	elongation factor Ts	30	13.5	J

gi|72080406|ref|YP_287464.1|	hypothetical protein MHP7448_0064	40	13.3	S
gi|72080408|ref|YP_287466.1|	excinuclease ABC subunit C	29	11	L
gi|72080409|ref|YP_287467.1|	molecular chaperone DnaK	969	36.5	O
gi|72080413|ref|YP_287471.1|	bacterial nucleoid DNA-binding protein	66	14.6	L
gi|72080417|ref|YP_287475.1|	elongation factor EF-2	134	22.2	K
gi|72080419|ref|YP_287477.1|	30S ribosomal protein S12	51	5.8	J
gi|72080424|ref|YP_287482.1|	NADH oxidase	478	16.5	R
gi|72080428|ref|YP_287486.1|	translocase	18	4.2	N
gi|72080434|ref|YP_287492.1|	hypothetical protein MHP7448_0092	28	12.5	S
gi|72080438|ref|YP_287496.1|	thiol peroxidase	107	40.2	O
gi|72080440|ref|YP_287498.1|	thioredoxin reductase	125	28.5	O
gi|72080443|ref|YP_287501.1|	ATP-dependent protease binding protein	37	5.5	O
gi|72080448|ref|YP_287506.1|	P97 paralog 1	344	19.3	S
gi|72080449|ref|YP_287507.1|	DNA gyrase subunit B	36	20	L
gi|72080451|ref|YP_287509.1|	6-phosphofructokinase	29	25.2	G
gi|72080454|ref|YP_287512.1|	adenine phosphoribosyltransferase	597	41.4	F
gi|72080455|ref|YP_287513.1|	pyruvate dehydrogenase E1-alpha subunit	1541	34.5	C
gi|72080456|ref|YP_287514.1|	pyruvate dehydrogenase	1185	62	C
gi|72080461|ref|YP_287519.1|	hypothetical protein MHP7448_0121	17	6.3	S
gi|72080466|ref|YP_287524.1|	pyruvate kinase	50	4.2	G

gi|72080471|ref|YP_287529.1|	50S ribosomal protein L21	32	17.2	J
gi|72080472|ref|YP_287530.1|	50S ribosomal protein L27	92	22.6	J
gi|72080473|ref|YP_287531.1|	lipase-esterase	80	20.8	R
gi|72080475|ref|YP_287533.1|	hexosephosphate transport protein	38	7.1	P
gi|72080476|ref|YP_287534.1|	L-lactate dehydrogenase	388	43.5	C
gi|72080487|ref|YP_287545.1|	hypothetical protein MHP7448_0148	33	8.3	S
gi|72080488|ref|YP_287546.1|	trigger factor	171	18.6	O
gi|72080489|ref|YP_287547.1|	hypothetical protein MHP7448_0150	18	4.7	S
gi|72080500|ref|YP_287558.1|	phosphopentomutase	16	6	G
gi|72080507|ref|YP_287565.1|	DNA-directed RNA polymerase alpha subunit	59	10.8	K
gi|72080508|ref|YP_287566.1|	50S ribosomal protein L11	93	32	J
gi|72080508|ref|YP_287566.1|	30S ribosomal protein S11	42	35.1	J
gi|72080509|ref|YP_287567.1|	30S ribosomal protein S13	67	14.6	J
gi|72080515|ref|YP_287573.1|	50S ribosomal protein L15	66	9	J
gi|72080519|ref|YP_287577.1|	30S ribosomal protein S8	78	16.8	J
gi|72080521|ref|YP_287579.1|	50S ribosomal protein L5	82	37.8	J
gi|72080522|ref|YP_287580.1|	50S ribosomal protein L24	87	15	J
gi|72080525|ref|YP_287583.1|	50S ribosomal protein L29	78	22	S
gi|72080528|ref|YP_287586.1|	50S ribosomal protein L22	50	15.3	J
gi|72080530|ref|YP_287588.1|	50S ribosomal protein L2	137	11.3	J

gi|72080532|ref|YP_287590.1|	50S ribosomal protein L4	47	12.1	J
gi|72080533|ref|YP_287591.1|	50S ribosomal protein L3	36	12.2	J
gi|72080537|ref|YP_287595.1|	protein P97	1550	26.3	S
gi|72080538|ref|YP_287596.1|	protein P102	575	25.1	S
gi|72080540|ref|YP_287598.1|	alanine--tRNA ligase	15	2.3	J
gi|72080545|ref|YP_287603.1|	cell division protein	130	11.7	O
gi|72080546|ref|YP_287604.1|	lysyl-tRNA synthetase	104	4.9	J
gi|72080556|ref|YP_287614.1|	lipoprotein	36	3.1	S
gi|72080560|ref|YP_287618.1|	ribonucleotide-diphosphate reductase alpha subunit	45	22.4	F
gi|72080562|ref|YP_287620.1|	ribonucleotide-diphosphate reductase beta subunit	33	6.5	F
gi|72080564|ref|YP_287622.1|	methylmalonate-semialdehyde dehydrogenase	346	38.2	C
gi|72080568|ref|YP_287626.1|	myo-inositol catabolism protein	91	11.7	G
gi|72080573|ref|YP_287631.1|	periplasmic sugar-binding proteins	51	27.4	G
gi|72080574|ref|YP_287632.1|	myo-inositol 2-dehydrogenase	20	3.2	R
gi|72080580|ref|YP_287638.1|	protein-export membrane protein	35	5	N
gi|72080583|ref|YP_287641.1|	hypothetical protein MHP7448_0244	54	9.9	S
gi|72080589|ref|YP_287647.1|	phosphopyruvate hydratase	141	35.4	G
gi|72080590|ref|YP_287648.1|	seryl-tRNA synthetase	31	7.5	J
gi|72080591|ref|YP_287649.1|	hypothetical protein MHP7448_0252	20	18	S
gi|72080593|ref|YP_287651.1|	lipoate-protein ligase A	14	6.9	H

gi|72080601|ref|YP_287659.1|	hypoxanthine-guanine phosphoribosyltransferase	30	34.3	F
gi|72080607|ref|YP_287665.1|	cation-transporting P-type ATPase	37	22.4	P
gi|72080611|ref|YP_287669.1|	P97 paralog 2	26	19	S
gi|72080613|ref|YP_287671.1|	phenylalanyl-tRNA synthetase beta subunit	43	14.8	J
gi|72080628|ref|YP_287686.1|	hypothetical protein MHP7448_0289	23	4	S
gi|72080634|ref|YP_287692.1|	30S ribosomal protein S6	51	16.2	J
gi|72080635|ref|YP_287693.1|	hypothetical protein MHP7448_0297	32	23.7	S
gi|72080644|ref|YP_287702.1|	ABC transporter ATP-binding protein	55	17.8	R
gi|72080647|ref|YP_287705.1|	NADH-dependent flavin oxidoreductase	28	24.6	C
gi|72080652|ref|YP_287710.1|	ABC transporter ATP-binding protein	15	3.2	R
gi|72080653|ref|YP_287711.1|	ABC transporter ATP-binding protein	55	18.3	R
gi|72080687|ref|YP_287745.1|	hypothetical protein MHP7448_0351	20	6.3	S
gi|72080688|ref|YP_287746.1|	hypothetical protein MHP7448_0352	29	13.7	S
gi|72080689|ref|YP_287747.1|	P60-like lipoprotein	44	4.1	S
gi|72080690|ref|YP_287748.1|	HIT-like protein	25	44.5	F
gi|72080692|ref|YP_287750.1|	hypothetical protein MHP7448_0356	29	15.2	S
gi|72080702|ref|YP_287760.1|	lipoprotein	151	16.3	S
gi|72080703|ref|YP_287761.1|	lipoprotein	28	20.3	S
gi|72080708|ref|YP_287766.1|	Lppt protein	115	17.8	S
gi|72080709|ref|YP_287767.1|	hypothetical protein MHP7448_0373	452	14.8	S

gi|72080711|ref|YP_287769.1|	PTS system enzyme IIB component	177	14.6	G
gi|72080713|ref|YP_287771.1|	hypothetical protein MHP7448_0377	434	30	S
gi|72080714|ref|YP_287772.1|	lipoprotein	17	3.5	S
gi|72080720|ref|YP_287778.1|	thioredoxin	272	44.5	O
gi|72080725|ref|YP_287783.1|	hypothetical protein MHP7448_0391	18	6.8	S
gi|72080726|ref|YP_287784.1|	S-adenosyl-methyltransferase	24	9.5	M
gi|72080735|ref|YP_287793.1|	asparaginyl-tRNA synthetase	69	18.5	J
gi|72080737|ref|YP_287795.1|	ATP-dependent helicase PcrA	49	5.2	L
gi|72080739|ref|YP_287797.1|	Holliday junction DNA helicase motor protein	15	18.1	L
gi|72080758|ref|YP_287816.1|	hypothetical protein MHP7448_0425	16	4.2	S
gi|72080761|ref|YP_287819.1|	transketolase	122	23.9	G
gi|72080771|ref|YP_287829.1|	3-hexulose-6-phosphate synthase	78	19.8	G
gi|72080786|ref|YP_287844.1|	acyl carrier protein phosphodiesterase	57	21.6	I
gi|72080791|ref|YP_287849.1|	50S ribosomal protein L1	19	7.4	J
gi|72080796|ref|YP_287854.1|	leucyl aminopeptidase	80	20.5	E
gi|72080798|ref|YP_287856.1|	hypothetical protein MHP7448_0466	133	15.7	S
gi|72080804|ref|YP_287862.1|	phosphoenolpyruvate-protein phosphotransferase	49	9.2	G
gi|72080810|ref|YP_287868.1|	ATP synthase subunit A	66	2.9	C
gi|72080813|ref|YP_287871.1|	hypothetical protein MHP7448_0482	58	27	S
gi|72080814|ref|YP_287872.1|	hypothetical protein MHP7448_0483	61	12.2	S

gi|72080820|ref|YP_287878.1|	hypothetical protein MHP7448_0489	38	14.2	S
gi|72080821|ref|YP_287879.1|	phosphoglycerate kinase	46	23.5	G
gi|72080825|ref|YP_287883.1|	mannose-6-phosphate isomerase	92	28	G
gi|72080827|ref|YP_287885.1|	putative p216 surface protein	1560	21.6	S
gi|72080828|ref|YP_287886.1|	p76 membrane protein precursor	1366	30.8	S
gi|72080837|ref|YP_287895.1|	dihydrolipoamide acetyltransferase	198	37.7	C
gi|72080839|ref|YP_287897.1|	acetate kinase	101	37.6	C
gi|72080840|ref|YP_287898.1|	phosphate acetyltransferase	441	28.7	C
gi|72080844|ref|YP_287902.1|	46K surface antigen precursor	900	26.5	S
gi|72080845|ref|YP_287903.1|	xylose ABC transporter ATP-binding protein	17	4.7	G
gi|72080852|ref|YP_287910.1|	oligoendopeptidase F	117	7.5	E
gi|72080854|ref|YP_287912.1|	elongation factor Tu	1229	48.3	J
gi|72080855|ref|YP_287913.1|	heat shock ATP-dependent protease	160	23.1	O
gi|72080859|ref|YP_287917.1|	DNA gyrase subunit A	14	5.8	L
gi|72080862|ref|YP_287920.1|	glucose-6-phosphate isomerase	49	5.8	G
gi|72080865|ref|YP_287923.1|	ribosome recycling factor	90	18.4	J
gi|72080873|ref|YP_287931.1|	spermidine/putrescine ABC transporter ATP-binding	27	14.6	E
gi|72080899|ref|YP_287957.1|	dihydrolipoamide dehydrogenase	47	4.2	C
gi|72080904|ref|YP_287962.1|	30S ribosomal protein S4	103	19	J
gi|72080909|ref|YP_287967.1|	5'-3' exonuclease	31	7.5	L

gi|72080914|ref|YP_287972.1|	transcription elongation factor NusA	41	16.2	K
gi|72080920|ref|YP_287978.1|	ATP binding protein	95	8.9	L
gi|72080923|ref|YP_287981.1|	phosphoglyceromutase	74	17.8	G
gi|72080929|ref|YP_287987.1|	hypothetical protein MHP7448_0601	15	9.7	S
gi|72080931|ref|YP_287989.1|	inorganic pyrophosphatase	42	10.3	C
gi|72080932|ref|YP_287990.1|	ABC transporter xylose-binding lipoprotein	372	15.2	R
gi|72080933|ref|YP_287991.1|	sugar ABC transporter ATP-binding protein	75	11.1	R
gi|72080944|ref|YP_288002.1|	DNA-directed RNA polymerase beta' subunit	15	2.7	K
gi|72080949|ref|YP_288007.1|	lipoprotein	61	15.5	S
gi|72080954|ref|YP_288012.1|	ABC transporter ATP-binding - Pr1-like	19	1.5	Q
gi|72080957|ref|YP_288015.1|	5'-nucleotidase precursor	20	7	F
gi|72080975|ref|YP_288033.1|	excinuclease ABC subunit B	26	20	L
gi|72080977|ref|YP_288035.1|	30S ribosomal protein S9	49	13.6	J
gi|72080982|ref|YP_288040.1|	glucose-inhibited division protein B	19	12.4	M
gi|72080983|ref|YP_288041.1|	prolipoprotein p65	939	35.7	S
gi|72080986|ref|YP_288044.1|	XAA-PRO aminopeptidase	89	23.8	E
gi|72080989|ref|YP_288047.1|	hypothetical protein MHP7448_0662	626	21.9	S
gi|72080990|ref|YP_288048.1|	adhesin like-protein P146	559	28.4	S
gi|72080999|ref|YP_288057.1|	valyl-tRNA synthetase	21	3.8	J

[1] CDS access number in the NCBI database (http://www.ncbi.nlm.nih.gov).

[2] Protein identification according to NCBI database (http://www.ncbi.nlm.nih.gov).
[3] MASCOT score is -10 x log(*P*), where *P* is the probability that the observed match is a random event.
[4] COG database functional classes: (J) Translation, ribosomal structure and biogenesis, (K) Transcription, (L) DNA replication, recombination and repair, (D) Cell division and chromosome partitioning, (O) Posttranslational modification, protein turnover, chaperones, (M) Cell envelope biogenesis, outer membrane, (N) Cell motility and secretion, (P) Inorganic ion transport and metabolism,(C) Energy production and conversion, (G) Carbohydrate transport and metabolism, (E) Amino acid transport and metabolism, (F) Nucleotide transport and metabolism, (H) Coenzyme metabolism, (I) Lipid metabolism, (Q) Secondary metabolites biosynthesis, transport and catabolism,(R) General function prediction only, and (S) Function unknown.
