# Supplementary material for: Comparative proteomic analysis of pathogenic and non-pathogenic strains from the swine pathogen Mycoplasma hyopneumoniae
Source: Proteome Sci. 2009 Dec 21;7:45. doi: 10.1186/1477-5956-7-45 (PMC2804596; doi:10.1186/1477-5956-7-45)
Supplement: Additional file 3 — Table S3 - Identification of 7448 strain proteins by LC-MS/MS. [file 1477-5956-7-45-S3.PDF]

**Table 3**

**Identification of 7422 strain proteins by LC-MS/MS**. Protein identified by a liquid chromatography (LC) separation (reversed-phase HPLC) coupled with a tandem mass spectrometry (MS/MS) by searching *M. hyopneumoniae* strain 7448 and J protein databases using MASCOT search engine.

| Accession number[1] | Protein description[2] | MASCOT score[3] | Sequence coverage (%) | COG[4] |
|---|---|---|---|---|
| gi\|72080345\|ref\|YP_287403.1\| | glucose-inhibited division protein A | 42 | 25.2 | D |
| gi\|72080347\|ref\|YP_287405.1\| | putative MgpA like-protein | 60 | 9.2 | R |
| gi\|72080348\|ref\|YP_287406.1\| | DHH family phosphoesterase | 74 | 14.1 | R |
| gi\|72080351\|ref\|YP_287409.1\| | hypothetical protein MHP7448_0009 | 75 | 24.8 | S |
| gi\|72080353\|ref\|YP_287411.1\| | heat shock protein | 118 | 12.8 | O |
| gi\|72080356\|ref\|YP_287414.1\| | fructose-bisphosphate aldolase | 326 | 13.6 | G |
| gi\|72080363\|ref\|YP_287421.1\| | ABC transporter ATP-binding protein | 14 | 4.5 | R |
| gi\|72080370\|ref\|YP_287428.1\| | amidase | 44 | 20.7 | J |
| gi\|72080377\|ref\|YP_287435.1\| | glyceraldehyde 3-phosphate dehydrogenase | 567 | 36.6 | G |
| gi\|72080379\|ref\|YP_287437.1\| | VACB-like ribonuclease II | 28 | 17.2 | K |
| gi\|72080395\|ref\|YP_287453.1\| | ATP synthase subunit B | 216 | 31 | C |
| gi\|72080397\|ref\|YP_287455.1\| | 30S ribosomal protein S2 | 533 | 34 | J |
| gi\|72080398\|ref\|YP_287456.1\| | elongation factor Ts | 70 | 30.7 | J |
| gi\|72080401\|ref\|YP_287459.1\| | DNA primase | 22 | 2 | L |
| gi\|72080409\|ref\|YP_287467.1\| | molecular chaperone DnaK | 543 | 24.5 | O |

| | | | | |
|---|---|---|---|---|
| gi\|72080413\|ref\|YP_287471.1\| | bacterial nucleoid DNA-binding protein | 38 | 57.3 | L |
| gi\|72080417\|ref\|YP_287475.1\| | elongation factor EF-2 | 364 | 27.6 | K |
| gi\|72080419\|ref\|YP_287477.1\| | 30S ribosomal protein S12 | 34 | 20.1 | J |
| gi\|72080424\|ref\|YP_287482.1\| | NADH oxidase | 1376 | 41.3 | R |
| gi\|72080425\|ref\|YP_287483.1\| | thymidinephosphorylase | 21 | 4 | F |
| gi\|72080426\|ref\|YP_287484.1\| | purine-nucleoside phosphorylase | 186 | 10.8 | F |
| gi\|72080428\|ref\|YP_287486.1\| | translocase | 30 | 25.3 | N |
| gi\|72080436\|ref\|YP_287494.1\| | hypothetical protein MHP7448_0094 | 38 | 20.7 | S |
| gi\|72080438\|ref\|YP_287496.1\| | thiolperoxidase | 13 | 14 | O |
| gi\|72080440\|ref\|YP_287498.1\| | thioredoxin reductase | 117 | 25.9 | O |
| gi\|72080441\|ref\|YP_287499.1\| | outer membrane protein - P95 | 17 | 1 | S |
| gi\|72080444\|ref\|YP_287502.1\| | triosephosphate isomerase | 35 | 9.1 | G |
| gi\|72080447\|ref\|YP_287505.1\| | protein P102 | 64 | 16.9 | S |
| gi\|72080448\|ref\|YP_287506.1\| | P97 paralog 2 | 303 | 21.2 | S |
| gi\|72080449\|ref\|YP_287507.1\| | DNA gyrase subunit B | 95 | 21.4 | L |
| gi\|72080451\|ref\|YP_287509.1\| | 6-phosphofructokinase | 68 | 13.4 | G |
| gi\|72080454\|ref\|YP_287512.1\| | adenine phosphoribosyltransferase | 495 | 47.9 | F |
| gi\|72080455\|ref\|YP_287513.1\| | pyruvate dehydrogenase E1-alpha subunit | 2274 | 39.3 | C |
| gi\|72080456\|ref\|YP_287514.1\| | pyruvate dehydrogenase | 2031 | 57.2 | C |
| gi\|72080461\|ref\|YP_287519.1\| | hypothetical protein MHP7448_0121 | 17 | 6 | S |

| | | | | |
|---|---|---|---|---|
| gi\|72080466\|ref\|YP_287524.1\| | pyruvate kinase | 224 | 28.8 | G |
| gi\|72080469\|ref\|YP_287527.1\| | aminopeptidase | 111 | 24.2 | G |
| gi\|72080471\|ref\|YP_287529.1\| | 50S ribosomal protein L21 | 90 | 16.2 | J |
| gi\|72080472\|ref\|YP_287530.1\| | 50S ribosomal protein L27 | 130 | 22.6 | J |
| gi\|72080473\|ref\|YP_287531.1\| | lipase-esterase | 52 | 27.9 | R |
| gi\|72080475\|ref\|YP_287533.1\| | hexosephosphate transport protein | 62 | 30.1 | P |
| gi\|72080476\|ref\|YP_287534.1\| | L-lactate dehydrogenase | 441 | 28.9 | C |
| gi\|72080477\|ref\|YP_287535.1\| | hypothetical protein MHP7448_0138 | 80 | 16.5 | S |
| gi\|72080487\|ref\|YP_287545.1\| | hypothetical protein MHP7448_0148 | 27 | 20.4 | S |
| gi\|72080488\|ref\|YP_287546.1\| | trigger factor | 347 | 22.5 | O |
| gi\|72080492\|ref\|YP_287550.1\| | guanylate kinase | 32 | 33 | F |
| gi\|72080495\|ref\|YP_287553.1\| | GTP-binding protein | 26 | 12.6 | R |
| gi\|72080500\|ref\|YP_287558.1\| | phosphopentomutase | 48 | 23.6 | G |
| gi\|72080507\|ref\|YP_287565.1\| | DNA-directed RNA polymerase alpha subunit | 296 | 14.2 | K |
| gi\|72080508\|ref\|YP_287566.1\| | 30S ribosomal protein S11 | 122 | 46.3 | J |
| gi\|72080509\|ref\|YP_287567.1\| | 30S ribosomal protein S13 | 44 | 21.9 | J |
| gi\|72080518\|ref\|YP_287576.1\| | 50S ribosomal protein L6 | 70 | 42.5 | J |
| gi\|72080519\|ref\|YP_287577.1\| | 30S ribosomal protein S8 | 31 | 8.4 | J |
| gi\|72080521\|ref\|YP_287579.1\| | 50S ribosomal protein L5 | 46 | 16.7 | J |
| gi\|72080522\|ref\|YP_287580.1\| | 50S ribosomal protein L24 | 53 | 13.1 | J |

| | | | | |
|---|---|---|---|---|
| gi\|72080525\|ref\|YP_287583.1\| | 50S ribosomal protein L29 | 78 | 22.1 | S |
| gi\|72080528\|ref\|YP_287586.1\| | 50S ribosomal protein L22 | 122 | 34.7 | J |
| gi\|72080530\|ref\|YP_287588.1\| | 50S ribosomal protein L2 | 113 | 27.3 | J |
| gi\|72080532\|ref\|YP_287590.1\| | 50S ribosomal protein L4 | 86 | 18.4 | J |
| gi\|72080533\|ref\|YP_287591.1\| | 50S ribosomal protein L3 | 116 | 12.6 | J |
| gi\|72080537\|ref\|YP_287595.1\| | protein P97 | 1199 | 26.9 | S |
| gi\|72080538\|ref\|YP_287596.1\| | protein P102 | 451 | 24.9 | S |
| gi\|72080539\|ref\|YP_287597.1\| | hypothetical protein MHP7448_0200 | 18 | 4 | S |
| gi\|72080540\|ref\|YP_287598.1\| | alanine--tRNAligase | 20 | 2 | J |
| gi\|72080545\|ref\|YP_287603.1\| | cell division protein | 335 | 20.7 | O |
| gi\|72080546\|ref\|YP_287604.1\| | lysyl-tRNA synthetase | 110 | 19.2 | J |
| gi\|72080547\|ref\|YP_287605.1\| | hydrolase of the HAD family | 22 | 5.4 | R |
| gi\|72080551\|ref\|YP_287609.1\| | oligopeptide ABC transporter system permease | 17 | 4.4 | E |
| gi\|72080554\|ref\|YP_287612.1\| | oligopeptideABCtransporterATP-bindingprotein | 17 | 7 | E |
| gi\|72080560\|ref\|YP_287618.1\| | ribonucleotide-diphosphate reductase alpha subunit | 38 | 7.7 | F |
| gi\|72080562\|ref\|YP_287620.1\| | ribonucleotide-diphosphate reductase beta subunit | 244 | 13.6 | F |
| gi\|72080564\|ref\|YP_287622.1\| | methylmalonate-semialdehyde dehydrogenase | 445 | 33.1 | C |
| gi\|72080568\|ref\|YP_287626.1\| | myo-inositol catabolism protein | 303 | 14.9 | G |
| gi\|72080573\|ref\|YP_287631.1\| | periplasmic sugar-binding proteins | 84 | 16.7 | G |
| gi\|72080582\|ref\|YP_287640.1\| | aspartyl-tRNA synthetase | 80 | 17.6 | J |

| | | | | |
|---|---|---|---|---|
| gi\|72080583\|ref\|YP_287641.1\| | hypothetical protein MHP7448_0244 | 46 | 9.9 | S |
| gi\|72080586\|ref\|YP_287644.1\| | TRSE-likeprotein | 23 | 3 | N |
| gi\|72080589\|ref\|YP_287647.1\| | phosphopyruvate hydratase | 224 | 20.8 | G |
| gi\|72080591\|ref\|YP_287649.1\| | hypothetical protein MHP7448_0252 | 29 | 16.9 | S |
| gi\|72080592\|ref\|YP_287650.1\| | triacylglycerol lipase | 38 | 12.8 | R |
| gi\|72080593\|ref\|YP_287651.1\| | lipoate-protein ligase A | 40 | 12.6 | H |
| gi\|72080596\|ref\|YP_287654.1\| | hypothetical protein MHP7448_0257 | 84 | 34.6 | S |
| gi\|72080597\|ref\|YP_287655.1\| | recombination protein RecR | 34 | 34.5 | L |
| gi\|72080601\|ref\|YP_287659.1\| | hypoxanthine-guanine phosphoribosyltransferase | 17 | 16.3 | F |
| gi\|72080605\|ref\|YP_287663.1\| | DNA ligase | 37 | 5.6 | L |
| gi\|72080611\|ref\|YP_287669.1\| | P97 paralog 2 | 56 | 11.6 | S |
| gi\|72080613\|ref\|YP_287671.1\| | phenylalanyl-tRNA synthetase beta subunit | 89 | 13.2 | J |
| gi\|72080618\|ref\|YP_287676.1\| | transcriptional regulator | 89 | 9.6 | K |
| gi\|72080619\|ref\|YP_287677.1\| | CTP synthetase | 30 | 19.8 | F |
| gi\|72080635\|ref\|YP_287693.1\| | hypothetical protein MHP7448_0297 | 39 | 32.6 | S |
| gi\|72080643\|ref\|YP_287701.1\| | ABCtransporterATP-bindingprotein | 18 | 1 | R |
| gi\|72080650\|ref\|YP_287708.1\| | glycine cleavage system H protein | 16 | 11.8 | E |
| gi\|72080682\|ref\|YP_287740.1\| | hypothetical protein MHP7448_0346 | 15 | 2 | S |
| gi\|72080688\|ref\|YP_287746.1\| | hypothetical protein MHP7448_0352 | 38 | 14.8 | S |
| gi\|72080689\|ref\|YP_287747.1\| | P60-like lipoprotein | 140 | 17.3 | S |

| | | | | |
|---|---|---|---|---|
| gi\|72080690\|ref\|YP_287748.1\| | HIT-like protein | 48 | 19.1 | F |
| gi\|72080702\|ref\|YP_287760.1\| | lipoprotein | 562 | 33.4 | S |
| gi\|72080703\|ref\|YP_287761.1\| | lipoprotein | 82 | 13.6 | S |
| gi\|72080708\|ref\|YP_287766.1\| | Lppt protein | 26 | 14.1 | S |
| gi\|72080709\|ref\|YP_287767.1\| | hypothetical protein MHP7448_0373 | 389 | 20.3 | S |
| gi\|72080711\|ref\|YP_287769.1\| | PTS system enzyme IIB component | 156 | 14.6 | G |
| gi\|72080713\|ref\|YP_287771.1\| | hypothetical protein MHP7448_0377 | 1003 | 50 | S |
| gi\|72080720\|ref\|YP_287778.1\| | thioredoxin | 351 | 44.5 | O |
| gi\|72080725\|ref\|YP_287783.1\| | hypothetical protein MHP7448_0391 | 18 | 6 | S |
| gi\|72080726\|ref\|YP_287784.1\| | S-adenosyl-methyltransferase | 14 | 9 | M |
| gi\|72080735\|ref\|YP_287793.1\| | asparaginyl-tRNA synthetase | 99 | 20.7 | J |
| gi\|72080737\|ref\|YP_287795.1\| | ATP-dependent helicase PcrA | 130 | 10.7 | L |
| gi\|72080761\|ref\|YP_287819.1\| | transketolase | 212 | 30.6 | G |
| gi\|72080771\|ref\|YP_287829.1\| | 3-hexulose-6-phosphate synthase | 129 | 22 | G |
| gi\|72080787\|ref\|YP_287845.1\| | acyl carrier protein phosphodiesterase | 57 | 25.4 | I |
| gi\|72080791\|ref\|YP_287849.1\| | 50S ribosomal protein L1 | 155 | 28.1 | J |
| gi\|72080792\|ref\|YP_287850.1\| | 50S ribosomal protein L11 | 117 | 26.2 | J |
| gi\|72080795\|ref\|YP_287853.1\| | hypothetical protein MHP7448_0463 | 17 | 2 | S |
| gi\|72080796\|ref\|YP_287854.1\| | leucyl aminopeptidase | 125 | 24.5 | E |
| gi\|72080798\|ref\|YP_287856.1\| | hypothetical protein MHP7448_0466 | 115 | 17.5 | S |

| | | | | |
|---|---|---|---|---|
| gi\|72080801\|ref\|YP_287859.1\| | ABC transporter atp-binding protein | 46 | 16.9 | R |
| gi\|72080804\|ref\|YP_287862.1\| | phosphoenolpyruvate-protein phosphotransferase | 260 | 17 | G |
| gi\|72080820\|ref\|YP_287878.1\| | hypothetical protein MHP7448_0489 | 83 | 20.7 | S |
| gi\|72080825\|ref\|YP_287883.1\| | mannose-6-phosphate isomerase | 126 | 17.6 | G |
| gi\|72080827\|ref\|YP_287885.1\| | putative p216 surface protein | 2622 | 34.4 | S |
| gi\|72080828\|ref\|YP_287886.1\| | p76 membrane protein precursor | 1885 | 27.3 | S |
| gi\|72080832\|ref\|YP_287890.1\| | oligopeptide ABC transporter ATP-binding protein | 20 | 5.2 | E |
| gi\|72080833\|ref\|YP_287891.1\| | oligopeptide ABC transporter ATP binding protein | 40 | 18.6 | E |
| gi\|72080837\|ref\|YP_287895.1\| | dihydrolipoamide acetyltransferase | 120 | 22.6 | C |
| gi\|72080838\|ref\|YP_287896.1\| | dihydrolipoamide dehydrogenase | 429 | 17.1 | C |
| gi\|72080839\|ref\|YP_287897.1\| | acetate kinase | 510 | 39.3 | C |
| gi\|72080840\|ref\|YP_287898.1\| | phosphate acetyltransferase | 823 | 39.7 | C |
| gi\|72080844\|ref\|YP_287902.1\| | 46K surface antigen precursor | 1275 | 39.9 | S |
| gi\|72080852\|ref\|YP_287910.1\| | oligoendopeptidase F | 193 | 10.6 | E |
| gi\|72080854\|ref\|YP_287912.1\| | elongation factor Tu | 1388 | 63.7 | J |
| gi\|72080855\|ref\|YP_287913.1\| | heat shock ATP-dependent protease | 263 | 25.6 | O |
| gi\|72080858\|ref\|YP_287916.1\| | deoxyribose-phosphate aldolase | 54 | 24.9 | F |
| gi\|72080859\|ref\|YP_287917.1\| | DNA gyrase subunit A | 27 | 17.9 | L |
| gi\|72080860\|ref\|YP_287918.1\| | methionine sulfoxide reductase B | 101 | 42.8 | O |
| gi\|72080862\|ref\|YP_287920.1\| | glucose-6-phosphate isomerase | 68 | 28 | G |

| | | | | |
|---|---|---|---|---|
| gi\|72080865\|ref\|YP_287923.1\| | ribosome recycling factor | 202 | 23 | J |
| gi\|72080909\|ref\|YP_287967.1\| | 5'-3' exonuclease | 54 | 14.6 | L |
| gi\|72080912\|ref\|YP_287970.1\| | translation initiation factor IF-2 | 25 | 21.7 | J |
| gi\|72080918\|ref\|YP_287976.1\| | tryptophanyl-tRNA synthetase | 124 | 39.3 | J |
| gi\|72080920\|ref\|YP_287978.1\| | ATP binding protein | 298 | 25 | L |
| gi\|72080923\|ref\|YP_287981.1\| | phosphoglyceromutase | 123 | 21 | G |
| gi\|72080932\|ref\|YP_287990.1\| | ABC transporter xylose-binding lipoprotein | 607 | 15.2 | R |
| gi\|72080933\|ref\|YP_287991.1\| | sugar ABC transporter ATP-binding protein | 87 | 25.8 | R |
| gi\|72080944\|ref\|YP_288002.1\| | DNA-directed RNA polymerase beta' subunit | 53 | 17 | K |
| gi\|72080945\|ref\|YP_288003.1\| | DNA-directed RNA polymerase beta subunit | 43 | 16.1 | K |
| gi\|72080946\|ref\|YP_288004.1\| | 50S ribosomal protein L7/L12 | 104 | 27.3 | J |
| gi\|72080949\|ref\|YP_288007.1\| | lipoprotein | 75 | 20.8 | S |
| gi\|72080952\|ref\|YP_288010.1\| | ABC transporter ATP-binding protein - Pr2 | 30 | 10.2 | Q |
| gi\|72080957\|ref\|YP_288015.1\| | 5'-nucleotidase precursor | 55 | 15.5 | F |
| gi\|72080959\|ref\|YP_288017.1\| | segregation and condensation protein A | 32 | 22.6 | L |
| gi\|72080974\|ref\|YP_288032.1\| | leucyl-tRNAsynthetase | 15 | 1 | J |
| gi\|72080977\|ref\|YP_288035.1\| | 30S ribosomal protein S9 | 91 | 10.6 | J |
| gi\|72080978\|ref\|YP_288036.1\| | 50S ribosomal protein L13 | 28 | 22.2 | J |
| gi\|72080983\|ref\|YP_288041.1\| | prolipoprotein p65 | 1485 | 27.6 | S |
| gi\|72080987\|ref\|YP_288045.1\| | hypothetical protein MHP7448_0660 | 30 | 16.9 | S |

| | | | | |
|---|---|---|---|---|
| gi\|72080989\|ref\|YP_288047.1\| | hypothetical protein MHP7448_0662 | 399 | 22.4 | S |
| gi\|72080990\|ref\|YP_288048.1\| | adhesin like-protein P146 | 533 | 27.7 | S |
| gi\|72080995\|ref\|YP_288053.1\| | transcription elongation factor | 188 | 30.6 | K |

[1] CDS access number in the NCBI database (http://www.ncbi.nlm.nih.gov).
[2] Protein identification according to NCBI database (http://www.ncbi.nlm.nih.gov).
[3] MASCOT score is -10 x log(*P*), where *P* is the probability that the observed match is a random event.
[4] COG database functional classes: (J) Translation, ribosomal structure and biogenesis, (K) Transcription, (L) DNA replication, recombination and repair, (D) Cell division and chromosome partitioning, (O) Posttranslational modification, protein turnover, chaperones, (M) Cell envelope biogenesis, outer membrane, (N) Cell motility and secretion, (P) Inorganic ion transport and metabolism,(C) Energy production and conversion, (G) Carbohydrate transport and metabolism, (E) Amino acid transport and metabolism, (F) Nucleotide transport and metabolism, (H) Coenzyme metabolism, (I) Lipid metabolism, (Q) Secondary metabolites biosynthesis, transport and catabolism,(R) General function prediction only, and (S) Function unknown.
