# Supplementary material for: Comparative proteomic analysis of pathogenic and non-pathogenic strains from the swine pathogen Mycoplasma hyopneumoniae
Source: Proteome Sci. 2009 Dec 21;7:45. doi: 10.1186/1477-5956-7-45 (PMC2804596; doi:10.1186/1477-5956-7-45)
Supplement: Additional file 4 — Table S4 - Identification of 7422 strain proteins by LC-MS/MS. [file 1477-5956-7-45-S4.PDF]

Table 4

The emPAI value comparison and differentially expressed proteins. The complete list of emPAI values for each protein from the three *M. hyopneumoniae* strains and differentially expressed proteins inferred by emPAI analysis.

Accession number[1]	Protein description[2]	emPAI value[3]		
		J	7448	7422
**Proteins identified only in strain J**				
gi|71893407|ref|YP_278853.1|	ATP synthase gamma chain	0.08	-	-
gi|71893826|ref|YP_279272.1|	ATP synthase subunit B	0.24	-	-
gi|71894005|ref|YP_279451.1|	ABC transporter ATP-binding protein P115-like	0.03	-	-
gi|71893706|ref|YP_279152.1|	amino acid permease	0.08	-	-
gi|71893574|ref|YP_279020.1|	serine hydroxymethyltransferase	0.10	-	-
gi|71893566|ref|YP_279012.1|	oligopeptide ABC transporter ATP-binding protein	0.20	-	-
gi|71893650|ref|YP_279096.1|	permease	0.06	-	-
gi|71893908|ref|YP_279354.1|	PTS system galactitol-specific enzyme IIB component	0.54	-	-
gi|71893760|ref|YP_279206.1|	methionyl-tRNA synthetase	0.05	-	-
gi|71893779|ref|YP_279225.1|	tRNA (5-methylaminomethyl-2-thiouridylate)-methyltransferase	0.08	-	-
gi|71893491|ref|YP_278937.1|	peptide chain release factor 1	0.12	-	-
gi|71893431|ref|YP_278877.1|	30S ribosomal protein S7	0.19	-	-
gi|71893621|ref|YP_279067.1|	phenylalanyl-tRNA synthetase alpha chain	0.19	-	-
gi|71893542|ref|YP_278988.1|	30S ribosomal protein S19	0.34	-	-

gi|71893476|ref|YP_278922.1|	50S ribosomal protein L20	0.38	-	-
gi|71893985|ref|YP_279431.1|	transcription antitermination protein NusG	0.15	-	-
gi|71893604|ref|YP_279050.1|	DNA polymerase III subunits gamma and tau	0.04	-	-
gi|71893972|ref|YP_279418.1|	DNA adenine methylase	0.05	-	-
gi|71893984|ref|YP_279430.1|	O-sialoglycoprotein endopeptidase	0.23	-	-
gi|71893937|ref|YP_279383.1|	glycerol-3-phosphate dehydrogenase	0.11	-	-
gi|71893796|ref|YP_279242.1|	hypothetical protein MHJ_0445	0.01	-	-
gi|71893793|ref|YP_279239.1|	hypothetical protein MHJ_0442	0.03	-	-
gi|71893768|ref|YP_279214.1|	hypothetical protein MHJ_0417	0.06	-	-
gi|71893816|ref|YP_279262.1|	hypothetical protein MHJ_0465	0.06	-	-
gi|71893920|ref|YP_279366.1|	hypothetical protein MHJ_0571	0.06	-	-
gi|71894021|ref|YP_279467.1|	hypothetical protein MHJ_0673	0.06	-	-
gi|71893568|ref|YP_279014.1|	hypothetical protein MHJ_0212	0.17	-	-
gi|71893639|ref|YP_279085.1|	hypothetical protein MHJ_0283	0.17	-	-
gi|71893472|ref|YP_278918.1|	hypothetical protein MHJ_0115	0.29	-	-
gi|71893572|ref|YP_279018.1|	ribonucleotide reductase stimulatory protein	0.43	-	-
	**Proteins identified only in strain 7448**			
gi|72080647|ref|YP_287705.1|	NADH-dependent flavin oxidoreductase	-	0.07	-
gi|72080873|ref|YP_287931.1|	spermidine/putrescine ABC transporter ATP-binding	-	0.06	-
gi|72080986|ref|YP_288044.1|	XAA-PRO aminopeptidase	-	0.08	-

gi|72080845|ref|YP_287903.1|	xylose ABC transporter ATP-binding protein	-	0.08	-
gi|72080999|ref|YP_288057.1|	valyl-tRNA synthetase	-	0.03	-
gi|72080904|ref|YP_287962.1|	30S ribosomal protein S4	-	0.48	-
gi|72080914|ref|YP_287972.1|	transcription elongation factor NusA	-	0.05	-
gi|72080975|ref|YP_288033.1|	excinuclease ABC subunit B	-	0.04	-
gi|72080408|ref|YP_287466.1|	excinuclease ABC subunit C	-	0.05	-
gi|72080373|ref|YP_287431.1|	lipoprotein signal peptidase	-	0.25	-
gi|72080607|ref|YP_287665.1|	cation-transporting P-type ATPase	-	0.03	-
gi|72080954|ref|YP_288012.1|	ABC transporter ATP-binding - Pr1-like	-	0.05	-
gi|72080652|ref|YP_287710.1|	ABC transporter ATP-binding protein	-	0.04	-
gi|72080653|ref|YP_287711.1|	ABC transporter ATP-binding protein	-	0.04	-
gi|72080383|ref|YP_287441.1|	GTP-binding protein Obg	-	0.07	-
gi|72080361|ref|YP_287419.1|	ABC transporter ATP-binding protein	-	0.08	-
gi|72080365|ref|YP_287423.1|	ABC transporter ATP-binding protein	-	0.08	-
gi|72080644|ref|YP_287702.1|	ABC transporter ATP-binding protein	-	0.08	-
gi|72080574|ref|YP_287632.1|	myo-inositol 2-dehydrogenase	-	0.08	-
gi|72080434|ref|YP_287492.1|	hypothetical protein MHP7448_0092	-	0.03	-
gi|72080692|ref|YP_287750.1|	hypothetical protein MHP7448_0356	-	0.05	-
gi|72080687|ref|YP_287745.1|	hypothetical protein MHP7448_0351	-	0.06	-
gi|72080758|ref|YP_287816.1|	hypothetical protein MHP7448_0425	-	0.11	-

gi|72080628|ref|YP_287686.1|	hypothetical protein MHP7448_0289	-	0.23	-
gi|72080489|ref|YP_287547.1|	hypothetical protein MHP7448_0150	-	0.24	-
	**Proteins identified only in strain 7422**			
gi|72080795|ref|YP_287853.1|	hypothetical protein MHP7448_0463	-	-	0.04
gi|72080974|ref|YP_288032.1|	leucyl-tRNA synthetase	-	-	0.04
gi|72080441|ref|YP_287499.1|	outer membrane protein-P95	-	-	0.04
gi|72080952|ref|YP_288010.1|	ABC transporter ATP-binding protein - Pr2	-	-	0.05
gi|72080945|ref|YP_288003.1|	DNA-directed RNA polymerase beta subunit	-	-	0.05
gi|72080987|ref|YP_288045.1|	hypothetical protein MHP7448_0660	-	-	0.05
gi|72080833|ref|YP_287891.1|	oligopeptide ABC transporter ATP binding protein	-	-	0.05
gi|72080447|ref|YP_287505.1|	protein P102	-	-	0.06
gi|72080401|ref|YP_287459.1|	DNA primase	-	-	0.07
gi|72080370|ref|YP_287428.1|	glutamyl-tRNA amidotransferase subunit A	-	-	0.07
gi|72080682|ref|YP_287740.1|	hypothetical protein MHP7448_0346	-	-	0.07
gi|72080832|ref|YP_287890.1|	oligopeptide ABC transporter ATP-binding protein	-	-	0.08
gi|72080801|ref|YP_287859.1|	ABC transporter atp-binding protein	-	-	0.09
gi|72080959|ref|YP_288017.1|	segregation and condensation protein A	-	-	0.12
gi|72080539|ref|YP_287597.1|	hypothetical protein MHP7448_0200	-	-	0.13
gi|72080347|ref|YP_287405.1|	MgpA like-protein	-	-	0.13
gi|72080495|ref|YP_287553.1|	GTP-binding protein	-	-	0.15

gi|72080363|ref|YP_287421.1|	ABC transporter ATP-binding protein	-	-	0.19
gi|72080643|ref|YP_287701.1|	ABC transporter ATP-binding protein	-	-	0.19
gi|72080492|ref|YP_287550.1|	guanylate kinase	-	-	0.22
gi|72080582|ref|YP_287640.1|	aspartyl-tRNA synthetase	-	-	0.24
gi|72080618|ref|YP_287676.1|	transcriptional regulator	-	-	0.34
gi|72080597|ref|YP_287655.1|	recombination protein RecR	-	-	0.49
gi|72080918|ref|YP_287976.1|	tryptophanyl-tRNA synthetase	-	-	0.63
gi|72080650|ref|YP_287708.1|	glycine cleavage system H protein	-	-	0.68
gi|72080860|ref|YP_287918.1|	methionine sulfoxide reductase B	-	-	1.44
	**Proteins identified in strains J and 7448**			
gi|71893589|ref|YP_279035.1|	protein-export membrane protein SecD	0.05	0.05	-
gi|72080556|ref|YP_287614.1|	lipoprotein	0.23	0.05	-
gi|71893954|ref|YP_279400.1|	inorganic pyrophosphatase	0.15	0.15	-
gi|71893827|ref|YP_279273.1|	ATP synthase subunit A	0.17	0.17	-
gi|72080899|ref|YP_287957.1|	dihydrolipoamide dehydrogenase	0.84	0.50	-
gi|71893838|ref|YP_279284.1|	phosphoglycerate kinase	0.33	0.15	-
gi|71893387|ref|YP_278833.1|	isoleucyl-tRNA synthetase	0.03	0.08	-
gi|71893599|ref|YP_279045.1|	seryl-tRNA synthetase	0.10	0.10	-
gi|71893643|ref|YP_279089.1|	30S ribosomal protein S6	0.24	0.11	-
gi|71893528|ref|YP_278974.1|	50S ribosomal protein L15	0.52	0.31	-

gi|71893769|ref|YP_279215.1|	Holliday junction DNA helicase motor protein	0.13	0.19	-
gi|71894003|ref|YP_279449.1|	glucose-inhibited division protein B	0.14	0.14	-
gi|71893457|ref|YP_278903.1|	ATP-dependent protease binding protein	0.12	0.12	-
gi|72080714|ref|YP_287772.1|	lipoprotein	1.61	0.05	-
gi|72080813|ref|YP_287871.1|	hypothetical protein MHP7448_0482	0.10	0.10	-
gi|72080929|ref|YP_287987.1|	hypothetical protein MHP7448_0601	0.10	0.10	-
gi|72080814|ref|YP_287872.1|	hypothetical protein MHP7448_0483	0.23	0.23	-
	**Proteins identified in strains J and 7422**			
gi|71893595|ref|YP_279041.1|	TRSE-like protein	0.03	-	0.03
gi|71893628|ref|YP_279074.1|	CTP synthetase	0.13	-	0.05
gi|71893614|ref|YP_279060.1|	DNA ligase	0.04	-	0.06
gi|71893934|ref|YP_279380.1|	translation initiation factor IF-2	0.10	-	0.07
gi|72080425|ref|YP_287483.1|	thymidine phosphorylase	0.17	-	0.07
gi|72080436|ref|YP_287494.1|	hypothetical protein MHP7448_0094	0.08	-	0.08
gi|72080554|ref|YP_287612.1|	oligopeptide ABC transporter ATP-binding protein	0.60	-	0.08
gi|72080487|ref|YP_287545.1|	hypothetical protein MHP7448_0148	0.14	-	0.10
gi|71893601|ref|YP_279047.1|	triacylglycerol lipase	0.16	-	0.11
gi|72080551|ref|YP_287609.1|	oligopeptide ABC transporter system permease	0.08	-	0.12
gi|71893879|ref|YP_279325.1|	deoxyribose-phosphate aldolase	0.29	-	0.14
gi|72080547|ref|YP_287605.1|	hydrolase of the HAD family	0.25	-	0.15

gi|71893458|ref|YP_278904.1|	triosephosphate isomerase	0.19	-	0.19
gi|71893999|ref|YP_279445.1|	50S ribosomal protein L13	0.31	-	0.31
gi|72080995|ref|YP_288053.1|	transcription elongation factor	0.19	-	0.41
gi|71893439|ref|YP_278885.1|	purine-nucleoside phosphorylase	0.63	-	0.47
gi|71893531|ref|YP_278977.1|	50S ribosomal protein L6	1.82	-	0.47
gi|71893968|ref|YP_279414.1|	50S ribosomal protein L7/L12	1.01	-	0.54
gi|72080469|ref|YP_287527.1|	aminopeptidase	0.42	-	0.59
gi|72080838|ref|YP_287896.1|	dihydrolipoamide dehydrogenase	0.15	-	0.90
gi|72080596|ref|YP_287654.1|	hypothetical protein MHP7448_0257	0.47	-	5.46
	**Proteins identified in strains 7448 and 7422**			
gi|72080690|ref|YP_287748.1|	HIT-like protein	-	0.27	0.26
gi|72080573|ref|YP_287631.1|	periplasmic sugar-binding proteins	-	0.16	0.37
gi|72080540|ref|YP_287598.1|	alanine--tRNA ligase	-	0.03	0.03
gi|72080419|ref|YP_287477.1|	30S ribosomal protein S12	-	0.22	0.22
gi|72080397|ref|YP_287455.1|	30S ribosomal protein S2	-	0.31	1.79
gi|72080379|ref|YP_287437.1|	VACB-like ribonuclease II	-	0.06	0.16
gi|72080507|ref|YP_287565.1|	DNA-directed RNA polymerase alpha subunit	-	0.22	0.72
gi|72080909|ref|YP_287967.1|	5'-3' exonuclease	-	0.24	0.14
gi|72080726|ref|YP_287784.1|	S-adenosyl-methyltransferase	-	0.10	0.10
gi|72080428|ref|YP_287486.1|	preprotein translocase SecA subunit	-	0.04	0.09

gi|72080475|ref|YP_287533.1|	hexosephosphate transport protein	-	0.09	0.19
gi|72080348|ref|YP_287406.1|	DHH family phosphoesterase	-	0.09	0.09
gi|72080406|ref|YP_287464.1|	hypothetical protein MHP7448_0064	-	0.02	0.02
gi|72080703|ref|YP_287761.1|	lipoprotein	-	0.09	0.04
gi|72080461|ref|YP_287519.1|	hypothetical protein MHP7448_0121	-	0.13	0.13
gi|72080525|ref|YP_287583.1|	50S ribosomal protein L29	-	0.36	0.62
	**Proteins identified in all strains**			
gi|71893408|ref|YP_278854.1|	ATP synthase subunit B	0.33	0.22	0.22
gi|71893521|ref|YP_278967.1|	30S ribosomal protein S11	0.59	0.23	0.59
gi|71893522|ref|YP_278968.1|	30S ribosomal protein S13	0.47	0.47	0.21
gi|71893532|ref|YP_278978.1|	30S ribosomal protein S8	0.23	0.52	0.23
gi|71893998|ref|YP_279444.1|	30S ribosomal protein S9	1.07	0.36	0.35
gi|71893787|ref|YP_279233.1|	3-hexulose-6-phosphate synthase	0.73	0.33	0.28
gi|72080844|ref|YP_287902.1|	46K surface antigen precursor	3.55	2.92	4.11
gi|71893807|ref|YP_279253.1|	50S ribosomal protein L1	0.87	0.19	0.83
gi|71893808|ref|YP_279254.1|	50S ribosomal protein L11	0.76	0.31	0.92
gi|71893543|ref|YP_278989.1|	50S ribosomal protein L2	0.66	0.22	0.84
gi|71893484|ref|YP_278930.1|	50S ribosomal protein L21	1.57	0.48	0.48
gi|71893541|ref|YP_278987.1|	50S ribosomal protein L22	0.40	0.16	0.58
gi|71893535|ref|YP_278981.1|	50S ribosomal protein L24	0.47	0.99	1.52

gi|71893485|ref|YP_278931.1|	50S ribosomal protein L27	0.61	0.33	1.34
gi|71893546|ref|YP_278992.1|	50S ribosomal protein L3	0.44	0.61	0.36
gi|71893545|ref|YP_278991.1|	50S ribosomal protein L4	0.79	0.62	0.42
gi|71893534|ref|YP_278980.1|	50S ribosomal protein L5	0.61	1.57	1.24
gi|72080957|ref|YP_288015.1|	5'-nucleotidase precursor	0.20	0.12	0.07
gi|71893464|ref|YP_278910.1|	6-phosphofructokinase	0.09	0.09	0.09
gi|71893955|ref|YP_279401.1|	ABC transporter xylose-binding lipoprotein	2.32	0.70	1.98
gi|71893856|ref|YP_279302.1|	acetate kinase	0.54	1.01	2.45
gi|71893803|ref|YP_279249.1|	acyl carrier protein phosphodiesterase	0.30	0.15	0.37
gi|71893467|ref|YP_278913.1|	adenine phosphoribosyltransferase	9.76	8.65	9.43
gi|72080990|ref|YP_288048.1|	adhesin like-protein P146	1.15	0.82	0.73
gi|71893765|ref|YP_279211.1|	asparaginyl-tRNA synthetase	0.15	0.25	0.06
gi|72080920|ref|YP_287978.1|	ATP binding protein	0.10	0.12	0.23
gi|71893767|ref|YP_279213.1|	ATP-dependent helicase PcrA	0.13	0.05	0.13
gi|71893426|ref|YP_278872.1|	bacterial nucleoid DNA-binding protein	4.33	1.60	0.83
gi|71893558|ref|YP_279004.1|	cell division protein	0.21	0.28	0.65
gi|71893854|ref|YP_279300.1|	dihydrolipoamide acetyltransferase	1.19	1.27	0.73
gi|71893880|ref|YP_279326.1|	DNA gyrase subunit A	0.12	0.03	0.03
gi|71893463|ref|YP_278909.1|	DNA gyrase subunit B	0.05	0.05	0.05
gi|71893966|ref|YP_279412.1|	DNA-directed RNA polymerase beta' subunit	0.04	0.02	0.04

gi|72080417|ref|YP_287475.1|	elongation factor EF-2	0.29	0.48	0.51
gi|71893411|ref|YP_278857.1|	elongation factor Ts	0.10	0.14	0.14
gi|71893875|ref|YP_279321.1|	elongation factor Tu	6.31	5.07	8.40
gi|71893373|ref|YP_278819.1|	fructose-bisphosphate aldolase	0.99	0.51	1.01
gi|71893883|ref|YP_279329.1|	glucose-6-phosphate isomerase	0.10	0.10	0.10
gi|71893362|ref|YP_278808.1|	glucose-inhibited division protein A	0.07	0.12	0.10
gi|71893390|ref|YP_278836.1|	glyceraldehyde 3-phosphate dehydrogenase	2.24	1.83	1.78
gi|71893876|ref|YP_279322.1|	heat shock ATP-dependent protease	0.26	0.19	0.34
gi|72080353|ref|YP_287411.1|	heat shock protein	1.93	0.28	1.24
gi|72080351|ref|YP_287409.1|	hypothetical protein MHP7448_0009	0.42	0.15	0.19
gi|72080477|ref|YP_287535.1|	hypothetical protein MHP7448_0138	0.03	0.17	0.08
gi|72080583|ref|YP_287641.1|	hypothetical protein MHP7448_0244	0.19	0.19	0.19
gi|72080591|ref|YP_287649.1|	hypothetical protein MHP7448_0252	0.51	0.23	0.23
gi|72080635|ref|YP_287693.1|	hypothetical protein MHP7448_0297	0.07	0.07	0.07
gi|72080688|ref|YP_287746.1|	hypothetical protein MHP7448_0352	0.06	0.10	0.06
gi|72080709|ref|YP_287767.1|	hypothetical protein MHP7448_0373	0.51	0.59	0.45
gi|72080713|ref|YP_287771.1|	hypothetical protein MHP7448_0377	5.74	2.34	6.21
gi|72080725|ref|YP_287783.1|	hypothetical protein MHP7448_0391	0.20	0.20	0.20
gi|72080798|ref|YP_287856.1|	hypothetical protein MHP7448_0466	0.27	0.27	0.11
gi|72080820|ref|YP_287878.1|	hypothetical protein MHP7448_0489	0.20	0.15	0.20

gi|72080989|ref|YP_288047.1|	hypothetical protein MHP7448_0662	1.10	0.87	0.42
gi|71893610|ref|YP_279056.1|	hypoxanthine-guanine phosphoribosyltransferase	0.61	0.16	0.25
gi|71893812|ref|YP_279258.1|	leucyl aminopeptidase	0.06	0.07	0.13
gi|72080473|ref|YP_287531.1|	lipase-esterase	0.27	0.27	0.16
gi|71893602|ref|YP_279048.1|	lipoate-protein ligase A	0.18	0.08	0.18
gi|72080702|ref|YP_287760.1|	lipoprotein	0.47	0.26	0.93
gi|72080949|ref|YP_288007.1|	lipoprotein	0.04	0.04	0.15
gi|71893489|ref|YP_278935.1|	L-lactate dehydrogenase	1.81	2.21	4.06
gi|72080708|ref|YP_287766.1|	Lppt protein	0.58	0.27	0.07
gi|71893559|ref|YP_279005.1|	lysyl-tRNA synthetase	0.18	0.08	0.08
gi|72080825|ref|YP_287883.1|	mannose-6-phosphate isomerase	0.34	0.19	0.33
gi|72080564|ref|YP_287622.1|	methylmalonate-semialdehyde dehydrogenase	0.15	0.80	1.41
gi|72080409|ref|YP_287467.1|	molecular chaperone DnaK	4.08	3.37	1.54
gi|71893579|ref|YP_279025.1|	myo-inositol catabolism protein	0.06	0.13	0.25
gi|71893437|ref|YP_278883.1|	NADH oxidase	4.49	2.28	9.77
gi|71893873|ref|YP_279319.1|	oligoendopeptidase F	0.30	0.23	0.41
gi|71893844|ref|YP_279290.1|	P216 surface protein	2.65	1.55	2.25
gi|72080689|ref|YP_287747.1|	P60-like lipoprotein	0.19	0.08	0.27
gi|72080828|ref|YP_287886.1|	p76 membrane protein precursor	2.80	1.62	1.93
gi|71893622|ref|YP_279068.1|	phenylalanyl-tRNA synthetase beta subunit	0.16	0.08	0.06

gi|71893857|ref|YP_279303.1|	phosphate acetyltransferase	5.59	1.58	4.18
gi|71893820|ref|YP_279266.1|	phosphoenolpyruvate-protein phosphotransferase	0.65	0.07	0.43
gi|71893944|ref|YP_279390.1|	phosphoglycero mutase	0.14	0.14	0.12
gi|71893513|ref|YP_278959.1|	phosphopentomutase	0.11	0.07	0.07
gi|71893598|ref|YP_279044.1|	phosphopyruvate hydratase	0.70	0.48	0.53
gi|72080983|ref|YP_288041.1|	prolipoprotein p65	4.04	1.99	3.55
gi|72080538|ref|YP_287596.1|	protein P102	1.26	0.91	0.84
gi|72080448|ref|YP_287506.1|	protein P97	0.19	1.97	1.81
gi|72080537|ref|YP_287595.1|	P97 paralog 1	0.22	0.25	0.42
gi|72080611|ref|YP_287669.1|	P97 paralog 2	0.04	0.03	0.04
gi|72080711|ref|YP_287769.1|	PTS system enzyme IIB component	1.36	0.54	2.61
gi|72080456|ref|YP_287514.1|	pyruvate dehydrogenase	18.34	10.49	37.38
gi|72080455|ref|YP_287513.1|	pyruvate dehydrogenase E1-alpha subunit	6.60	7.50	15.18
gi|71893479|ref|YP_278925.1|	pyruvate kinase	0.47	0.09	0.47
gi|71893571|ref|YP_279017.1|	ribonucleotide-diphosphate reductase alpha subunit	0.20	0.04	0.06
gi|72080562|ref|YP_287620.1|	ribonucleotide-diphosphate reductase beta subunit	0.12	0.12	0.86
gi|71893886|ref|YP_279332.1|	ribosome recycling factor	0.74	0.57	1.13
gi|71893956|ref|YP_279402.1|	sugar ABC transporter ATP-binding protein	0.06	0.06	0.06
gi|71893452|ref|YP_278898.1|	thiol peroxidase	1.29	1.01	0.18
gi|71893733|ref|YP_279179.1|	thioredoxin	3.41	5.27	7.41

gi|71893454|ref|YP_278900.1|	thioredoxin reductase	0.32	0.46	0.73
gi|71893777|ref|YP_279223.1|	transketolase	0.40	0.15	0.60
gi|71893501|ref|YP_278947.1|	trigger factor	0.49	0.60	1.02

[1] CDS access number in the NCBI database (http://www.ncbi.nlm.nih.gov).

[2] Protein identification according to NCBI database (http://www.ncbi.nlm.nih.gov).

[3] The exponentially modified protein abundance index value (emPAI) is the transformed ratio of the number of experimentally observed peptides to the total number of peptides calculated by MASCOT software (Matrix Science, London, UK). The proteins with a difference of two fold or more in emPAI (marked in gray) were considered differentially expressed.
