# Supplementary figures and images for: Comparative proteomic analysis of pathogenic and non-pathogenic strains from the swine pathogen Mycoplasma hyopneumoniae
Source: Proteome Sci. 2009 Dec 21;7:45. doi: 10.1186/1477-5956-7-45 (PMC2804596; doi:10.1186/1477-5956-7-45)

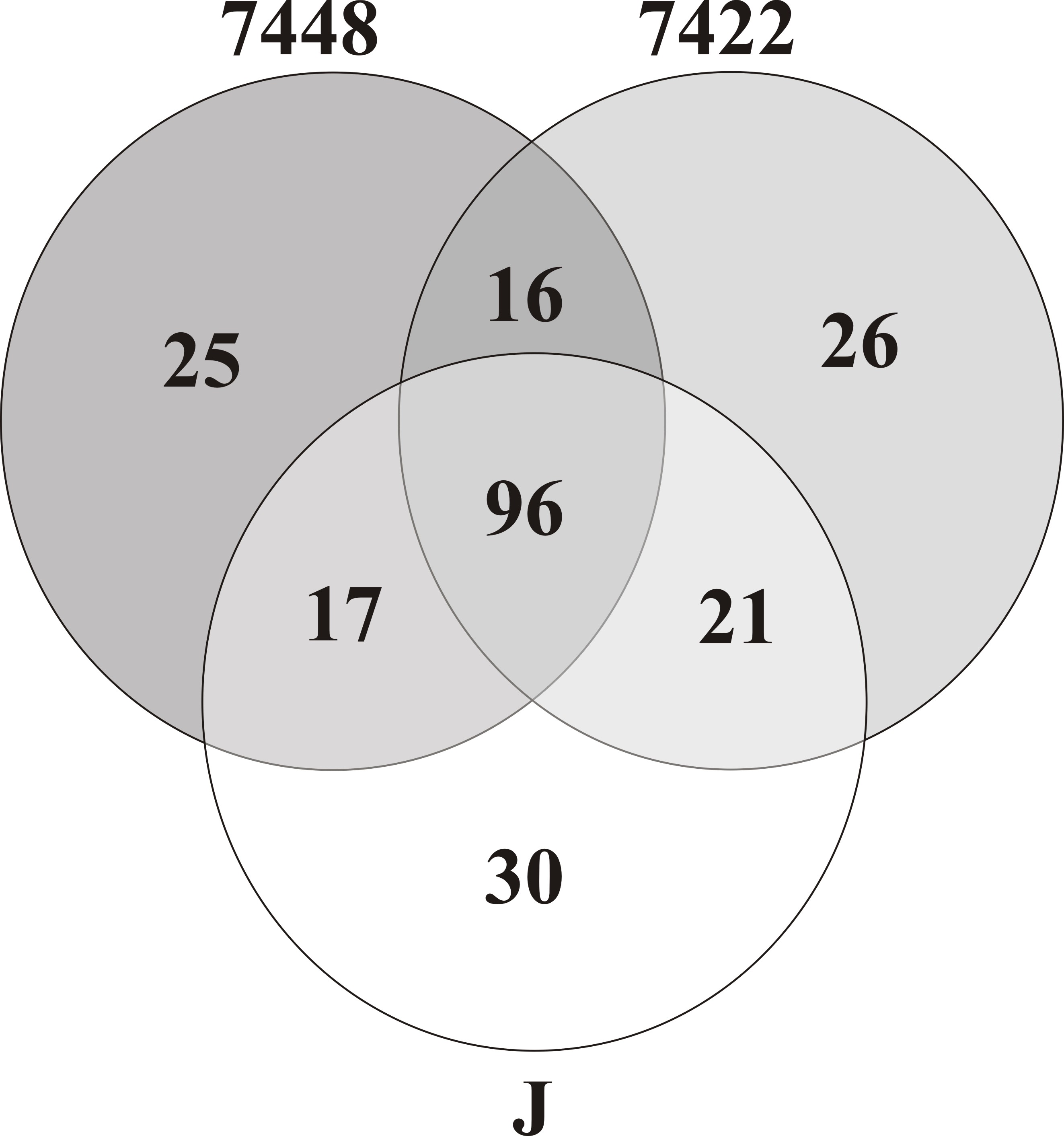

Supplement: Additional file 5 — Figure S1 - Venn diagram of the protein sets. Venn diagram obtained from the comparison of LC-MS/MS identified protein repertoires of M. hyopneumoniae strains J, 7448, and 7422. [file 1477-5956-7-45-S5.JPEG]

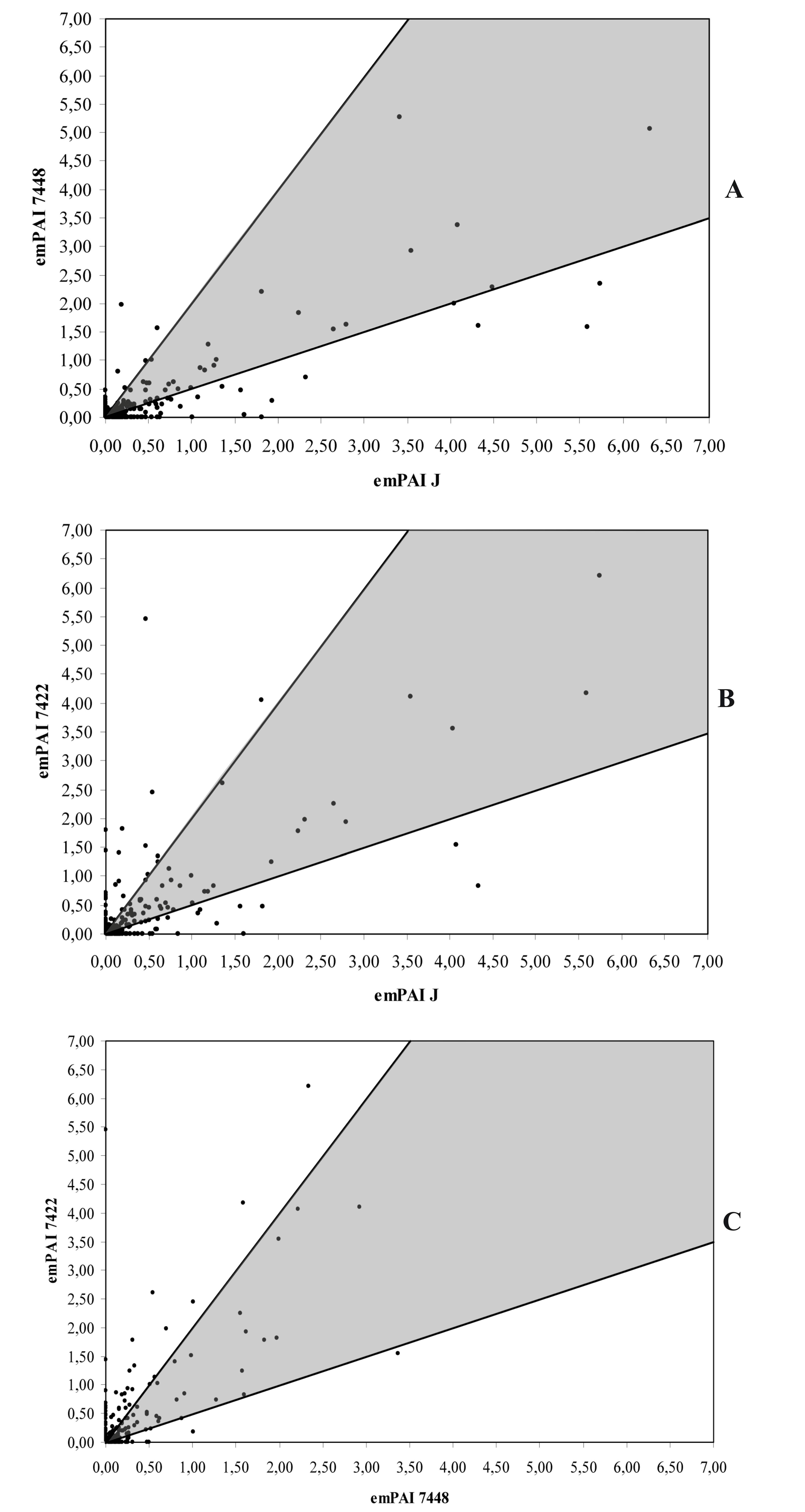

Supplement: Additional file 6 — Figure S2 - Comparative analyses of M. hyopneumoniae strains J, 7448, and 7422 LC-MS/MS proteomes based on emPAI relative abundance. (A-C) Bivariant plots of emPAI values (average of three independent LC-MS/MS analysis, with less than 5% difference between than) of proteins from: strain J against strain 7448 (A); strain J against strain 7422 (B); and strain 7448 against strain 7422 (C). Plotted protein emPAI values are in Table S1 (Additional File). Proteins outside the V-shaped shaded area (marked in gray) were assumed to be overexpressed in the strain whose emPAI values are assigned to the proximal axis. For overexpression assumption, a two-fold or higher difference between the emPAI values for a given protein in the two compared strains was required. [file 1477-5956-7-45-S6.JPEG]

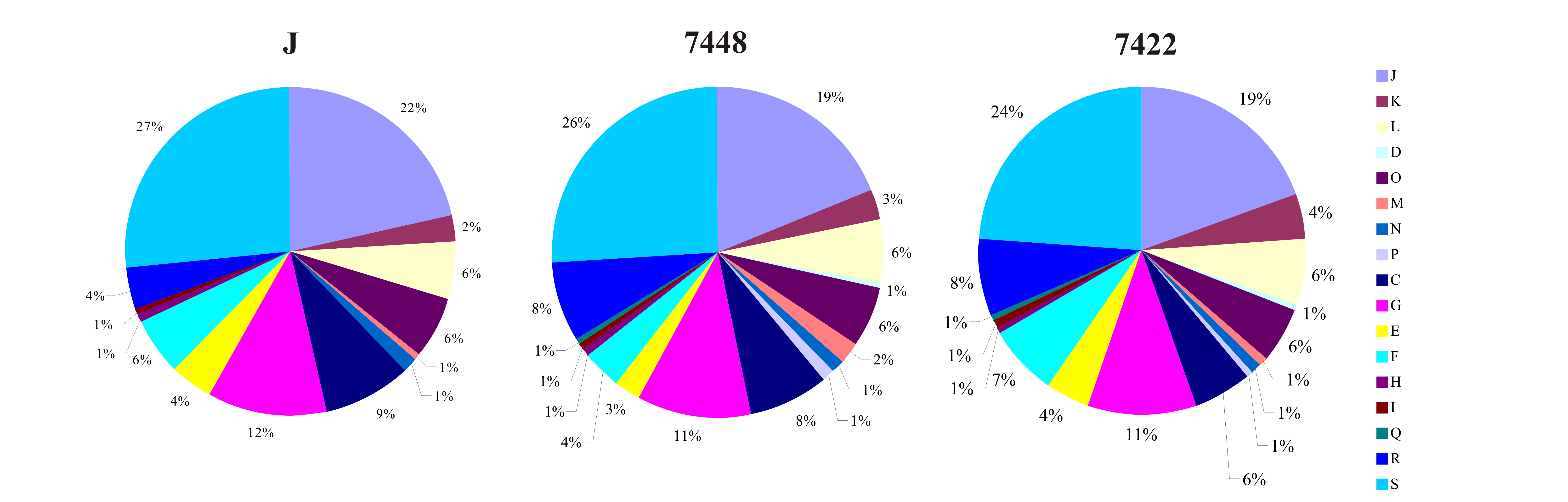

Supplement: Additional file 7 — Figure S3 - COG functional classification of identified proteins from M. hyopneumoniae strains J, 7448, and 7422. Schematic representations of identified proteins belonging to the COG functional classes as follows: Major class Information storage and processes: (J) Translation, ribosomal structure and biogenesis, (K) Transcription, (L) DNA replication, recombination, and repair; Major class Cellular processes: (D) Cell division and chromosome partitioning, (O) Post-translational modification, protein turnover, and chaperones, (M) Cell envelope biogenesis, outer membrane, (N) Cell motility and secretion, (P) Inorganic ion transport and metabolism; Major class Metabolism: (C) Energy production and conversion, (G) Carbohydrate transport and metabolism, (E) Amino acid transport and metabolism, (F) Nucleotide transport and metabolism, (H) Coenzyme metabolism, (I) Lipid metabolism, (Q) Secondary metabolites biosynthesis, transport, and catabolism; Major class Poorly characterised: (R) General function prediction only, (S) Function unknown. [file 1477-5956-7-45-S7.JPEG]
